# Identification of Residues in the Lipopolysaccharide ABC Transporter That Coordinate ATPase Activity with Extractor Function

**DOI:** 10.1128/mBio.01729-16

**Published:** 2016-10-18

**Authors:** Brent W. Simpson, Tristan W. Owens, Matthew J. Orabella, Rebecca M. Davis, Janine M. May, Sunia A. Trauger, Daniel Kahne, Natividad Ruiz

**Affiliations:** a Department of Microbiology, The Ohio State University, Columbus, Ohio, USA; b Department of Chemistry and Chemical Biology, Harvard University, Cambridge, Massachusetts, USA; c FAS Small Molecule Mass Spectrometry Facility, Harvard University, Cambridge, Massachusetts, USA; d Department of Molecular and Cellular Biology, Harvard University, Cambridge, Massachusetts, USA; e Department of Biological Chemistry and Molecular Pharmacology, Harvard Medical School, Boston, Massachusetts, USA

## Abstract

The surface of most Gram-negative bacteria is covered with lipopolysaccharide (LPS), creating a permeability barrier against toxic molecules, including many antimicrobials. To assemble LPS on their surface, Gram-negative bacteria must extract newly synthesized LPS from the inner membrane, transport it across the aqueous periplasm, and translocate it across the outer membrane. The LptA to -G proteins assemble into a transenvelope complex that transports LPS from the inner membrane to the cell surface. The Lpt system powers LPS transport from the inner membrane by using a poorly characterized ATP-binding cassette system composed of the ATPase LptB and the transmembrane domains LptFG. Here, we characterize a cluster of residues in the groove region of LptB that is important for controlling LPS transport. We also provide the first functional characterization of LptFG and identify their coupling helices that interact with the LptB groove. Substitutions at conserved residues in these coupling helices compromise both the assembly and function of the LptB_2_FG complex. Defects in LPS transport conferred by alterations in the LptFG coupling helices can be rescued by changing a residue in LptB that is adjacent to functionally important residues in the groove region. This suppression is achieved by increasing the ATPase activity of the LptB_2_FG complex. Taken together, these data identify a specific binding site in LptB for the coupling helices of LptFG that is responsible for coupling of ATP hydrolysis by LptB with LptFG function to achieve LPS extraction.

## IMPORTANCE

Lipopolysaccharide (LPS) is synthesized at the cytoplasmic membrane of Gram-negative bacteria and transported across several compartments to the cell surface, where it forms a barrier that protects these organisms from antibiotics. The LptB_2_FG proteins form an ATP-binding cassette (ABC) transporter that uses energy from ATP hydrolysis in the cytoplasm to facilitate extraction of LPS from the outer face of the cytoplasmic membrane prior to transport to the cell surface. How ATP hydrolysis is coupled with LPS release from the membrane is not understood. We have identified residues at the interface between the ATPase and the transmembrane domains of this heteromeric ABC complex that are important for LPS transport, some of which coordinate ATPase activity with LPS release.

## INTRODUCTION

The cell envelope of Gram-negative bacteria contains two lipid membranes with differing compositions that are separated by an aqueous compartment called the periplasm (1). Whereas the inner membrane (IM) is a phospholipid bilayer, the outer membrane (OM) of most Gram-negative bacteria has phospholipids in its inner leaflet and lipopolysaccharides (LPS) in its outer leaflet (1, 2). LPS molecules are tightly packed and anchored to the OM by a lipid moiety (lipid A) while displaying an elaborate network of sugars (core oligosaccharides and O antigen) on the cell surface (1, 3). Because of these structural features, LPS confers unique permeability characteristics that make the OM impermeable to many hydrophobic compounds (3). As a result, typical Gram-negative bacteria are naturally resistant to many hydrophobic antibiotics. Therefore, understanding LPS biogenesis might lead to strategies that could sensitize these bacteria to those antibiotics that cannot cross their OM.

**FIG 1:**
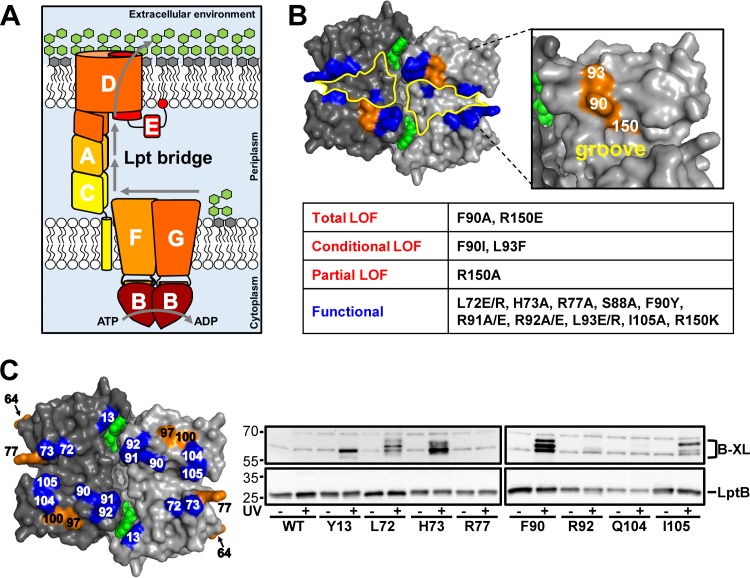
LptB contains a cluster of residues important for LPS transport that form interprotein cross-links. (A) Model of LPS transport by the Lpt system. (B) At the top is a surface representation of an LptB dimer (Protein Data Bank accession no. 4P33; membrane-facing view) with monomers in different shades of gray and ATP in green. The groove region (outlined in yellow) at the membrane interface of LptB contains residues important for LptB function (orange). Substitutions at blue residues do not cause defects when present in pET23/42LptB in strains carrying a chromosomal Δ*lptB* allele. At the bottom is a tabular summary of the functional classifications of *lptB* alleles encoding groove variants and the amino acid substitutions tested. Functional, behaves like WT *lptB*; partial LOF (loss of function), increases OM permeability to antibiotics; conditional LOF, cannot complement a chromosomal Δ*lptB* allele in rich medium but can do so in minimal medium; total LOF, cannot complement a chromosomal Δ*lptB* allele. (C) At the left is the structure of an LptB dimer showing residues (position numbers in LptB shown) that, when replaced with *p*BPA, form (blue) or do not form (orange) UV-dependent cross-links*.* ATP is green. At the right are immunoblot assays of *p*BPA-containing LptB variants (substitution sites are shown below the lanes) encoded by pET23/42LptB treated (+) or not treated (−) with UV. At the top are cross-linking adducts (B-XL), and at the bottom is un-cross-linked LptB. The WT is strain NR3877, which contains no *p*BPA substitutions. LptB^R77pBPA^ is a variant with no detectable cross-links. See Fig. S3A in the supplemental material for heightened contrast to enhance bands corresponding to cross-links when LptB contains *p*BPA at positions R92 and Q104. The values to the left are molecular sizes in kilodaltons.

Assembly of LPS at the cell surface requires that after it is synthesized at the IM (4), LPS is extracted from the outer leaflet of the IM, transported through the aqueous periplasm, and translocated across the OM. This transport of LPS across the cell envelope is mediated by the Lpt system (5), which comprises seven different proteins (LptA to -G) that assemble into a complex spanning from the cytoplasm to the OM (Fig. 1A) (6, 7). At the IM, the LptB_2_FG subcomplex is an unusual ATP-binding cassette (ABC) transporter that powers LPS transport by using the cytoplasmic ATPase LptB (8–12). In the periplasm, the OstA-like domains of the bitopic IM protein LptC, the periplasmic protein LptA, and the OM protein LptD interact with LPS (8, 13–17). At the OM, the LptDE translocon delivers LPS to the cell surface (18–20). Although we still lack important details of the architecture and mechanism of function of the Lpt system, a model, known as the PEZ model, has been proposed to explain how Lpt transports LPS from the IM to the cell surface (5). In this model, ATP hydrolysis by LptB is used to extract newly synthesized LPS molecules from the IM and to load them onto the Lpt complex. Once on the periplasmic Lpt bridge, LPS molecules travel as a stream from LptC to LptA and then to LptD; loading new LPS molecules onto the Lpt bridge pushes the LPS stream toward the OM. Then, when LPS arrives at the end of the periplasmic domain of LptD, the LptDE translocon inserts it directly into the outer leaflet of the OM, possibly through a lateral opening in the LptD barrel (14, 21–23).

Like other ABC transporters, LptB_2_FG is composed of two nucleotide-binding domains (LptB_2_) and two transmembrane domains (LptFG) (12, 24). However, unlike most ABC transporters, which transport substrates across membranes, LptB_2_FG extracts a lipid from a membrane. At present, we do not understand how LptB_2_FG functions and LptFG remain mostly uncharacterized (9). In fact, it is unknown which IM Lpt protein(s), LptC, LptF, or LptG, extracts LPS from the outer leaflet of the IM, although a recent study has reported the isolation of mutants lacking LptC (25). Therefore, LptFG must either directly extract LPS from the IM or indirectly promote LPS extraction by controlling LptC (or possibly LptA in its absence [25]), which is anchored to the IM and binds LPS (8, 16). In either model, LptFG must use physical interactions with the ATPase LptB to link ATP hydrolysis in the cytoplasm with LPS extraction in the periplasmic leaflet of the IM. Therefore, identification of functionally relevant contacts between LptB and LptFG is crucial to understanding LPS transport.

Most of the interface between LptB and LptFG remains uncharacterized. We previously proposed that grooves in LptB dimers interact with LptFG (10). No domains in LptFG that interact with LptB have been identified; however, on the basis of knowledge from other ABC transporters, we anticipate that a short coupling helix in each LptF and LptG inserts into the groove of an LptB monomer (26). In typical ABC transporters, the ATPase dimer undergoes conformational changes during the ATPase cycle (nucleotide binding, hydrolysis, and exchange), and the coupling helices are thought to transmit these changes to the transmembrane domains to facilitate substrate transport across the membrane (24, 26).

Here, we sought to define critical interfacial residues in LptB and LptFG that interact to coordinate ATP hydrolysis with LPS extraction. We characterized a cluster of residues centered at F90 in the groove of LptB that is important for LPS transport and demonstrated that F90 directly contacts LptFG. We also identified and functionally characterized the coupling helices of LptFG that interact with LptB. Furthermore, selecting for suppressors of defects in the coupling helices established a functional connection between the coupling helices of LptFG and the functional cluster of residues in the groove region of LptB. We showed that one of these residues can differentially alter the ATPase activity of the LptB_2_FG complex, depending on the functional status of the coupling helices of LptFG.

## RESULTS

### Identification of a cluster of residues in the groove of LptB_2_ that is critical for LPS transport.

We wanted to identify residues in LptB that are critical for coordinating ATPase activity with LPS extraction from the IM. We reasoned that some of these residues could be localized at its interface with LptFG. Residue F90 in the LptB groove is essential for the assembly of the LptB_2_FGC complex in *Escherichia coli* (10), suggesting that the groove of LptB forms part of an interacting surface with LptFG. To further characterize this groove region, we generated plasmid-carried mutant alleles of *lptB* that alter side chains flanking this groove and assessed their abilities to complement the loss of the wild-type (WT) chromosomal *lptB* allele in rich (LB) and minimal media since slow-growth conditions can suppress severe LPS transport defects (27). We also monitored the functional status of the Lpt system in the *lptB* mutants by analyzing the permeability of their OM to hydrophobic antibiotics.

Many residues in the groove of LptB could be changed without affecting LPS transport (Fig. 1B). However, substitutions within a cluster of residues comprising F90, L93, and R150 altered LPS transport to various degrees, depending on the position and nature of the change (Fig. 1B; see Fig. S1 in the supplemental material). Immunoblot analysis showed that although some substitutions (F90I, L93F, and R150E) caused a reduction in LptB levels, they were still present at levels greater than those produced by chromosomal *lptB* (see Fig. S1B in the supplemental material). Therefore, the defects observed were not simply the result of reduced LptB levels.

We wondered if this cluster of residues constitutes an LptFG binding site that is important for the function of LptB. Residues F90 and R150 are highly conserved among LptB orthologs, whereas L93 is less conserved (see Fig. S2 in the supplemental material). Although the aromaticity of F90 is required for the assembly of the LptB_2_FGC complex (10), here we found that *lptB*(F90I) haploid cells are viable under slow-growth conditions and exhibit severe OM permeability defects (Fig. 1B; see S1A in the supplemental material). We found similar defects when L93, which is positioned just behind F90, was replaced with phenylalanine (Fig. 1B; see Fig. S1A). This was surprising because changing L93 to charged residues (glutamate and arginine) did not cause defects (Fig. 1B). Given that the aromatic nature of F90 in LptB is crucial for the assembly of the LptB_2_FGC complex (10) and L93 is adjacent to F90, we propose that LptB^L93F^ is defective because L93F aligns through π stacking with F90, preventing F90 from mediating a critical interaction(s) with LptFG (see below). Substitutions in LptB at residue R150, which is located underneath F90 at the bottom of the groove, also affected LPS transport (Fig. 1B). Specifically, R150A and R150E rendered LptB partially and totally nonfunctional, respectively. Because an R150K substitution did not cause any detectable defects, we propose that the positive charge of R150 mediates ionic interactions important for LPS transport. Together, these results suggest that F90, L93, and R150 represent a locus in the groove of LptB that might mediate direct interactions with its transmembrane partners LptFG.

### Residue 90 in LptB directly contacts LptFG.

We wondered if the essential residues in the LptB groove region interact directly with LptFG. To look for interactions *in vivo*, we carried out site-specific cross-linking by incorporating the UV cross-linkable amino acid *p*-benzoyl phenylalanine (*p*BPA) at specific sites in LptB (28, 29). Only strains carrying *p*BPA-encoding alleles that complemented a chromosomal *lptB* deletion were tested for the appearance of cross-linked products, which were identified as UV-dependent, mass-upshifted bands in LptB immunoblot assays. Guided by our functional data (Fig. 1B) and the fact that *p*BPA is an aromatic amino acid, we chose to replace residue F90 with *p*BPA first. UV treatment of cells producing LptB^F90pBPA^, followed by LptB immunoblot analysis, revealed three strong, UV-dependent LptB^F90pBPA^ cross-links (Fig. 1C). The masses of these adducts were similar to those expected for LptB-LptF/G cross-links (ca. 67 kDa), further suggesting that F90 interacts directly with LptFG (see below).

We asked if other residues flanking the groove of LptB would show *in vivo* cross-linking. Since the LptB groove can tolerate nonconservative substitutions at various sites (Fig. 1B), we introduced *p*BPA at additional positions in LptB. As shown for F90, multiple UV-dependent cross-links appeared when *p*BPA was introduced at positions L72, H73, and I105 in LptB, while *p*BPA substitutions at Y13, R92, and Q104 yielded a single UV-dependent cross-linked product (Fig. 1C; see Fig. S3A in the supplemental material). However, none of these variants produced cross-links as intense as those observed for LptB^F90pBPA^. Finally, *p*BPA substitutions at positions located away from the groove (D97 and M100) or facing the cytoplasm (D64 and R77) did not yield UV-dependent bands (Fig. 1C; see Fig. S3B). Thus, although several positions in the LptB groove might interact with LptFG, F90 is the most efficient of these cross-linking sites.

Several lines of evidence establish that the three strong UV-dependent bands observed for LptB^F90pBPA^ correspond to cross-links of LptB with itself and with LptF or LptG. In our earlier experiment (Fig. 1C), LptB^F90pBPA^ was produced from a plasmid while LptFG were produced from the chromosome. When we performed *in vivo* cross-linking experiments with a strain carrying pBAD18LptFG3 to increase the levels of LptFG, we detected only two strong adducts with the LptB antiserum; these cross-links could also be detected with LptF and LptG antisera (see Fig. S4 in the supplemental material). Under these new conditions, there was only a trace of the third lower-mass cross-link we had readily detected when LptFG were only produced from the chromosome (compare Fig. S4 in the supplemental material and Fig. 1C). We propose that this lower-mass band corresponds to the noncanonical LptB dimer described in structural studies (10). This dimer, in which LptB monomers interact with each other at their respective membrane-facing surfaces, might form when levels of LptB are higher than those of LptFG.

### Identification of the coupling helices of LptFG that mediate interactions with the groove region of LptB.

In ABC transporters, the grooves of nucleotide-binding domains accommodate helical segments known as coupling helices from their transmembrane partners (24, 26). These domains are essential for function and have been proposed to coordinate ATPase activity and transport. In LptFG, coupling helices likely coordinate LptB ATPase activity with LPS extraction. Many coupling helices of ABC importers contain an EAA motif (30, 31) that is not present in LptFG. Nonetheless, we thought it likely that the coupling helices would be conserved among LptFG homologs in a cytoplasmic domain. When we compared distant LptFG homologs, we identified the consensus sequence Φ----EΦ-ΦΦ---G (Φ represents a hydrophobic amino acid, and - represents any amino acid) in the predicted cytoplasmic loop located between transmembrane helices 2 and 3 (see Fig. S5 in the supplemental material). The glutamate and glycine in this consensus sequence are the only two residues absolutely conserved among the representative LptFG homologs (see Fig. S5). From the alignment of this cytoplasmic loop in the *E. coli* LptF and LptG sequences, we predicted that the coupling helices might encompass the 16 residues centered on the conserved glutamates (residues 77 to 92 in LptF and 81 to 96 in LptG; see Fig. S5).

**FIG 2:**
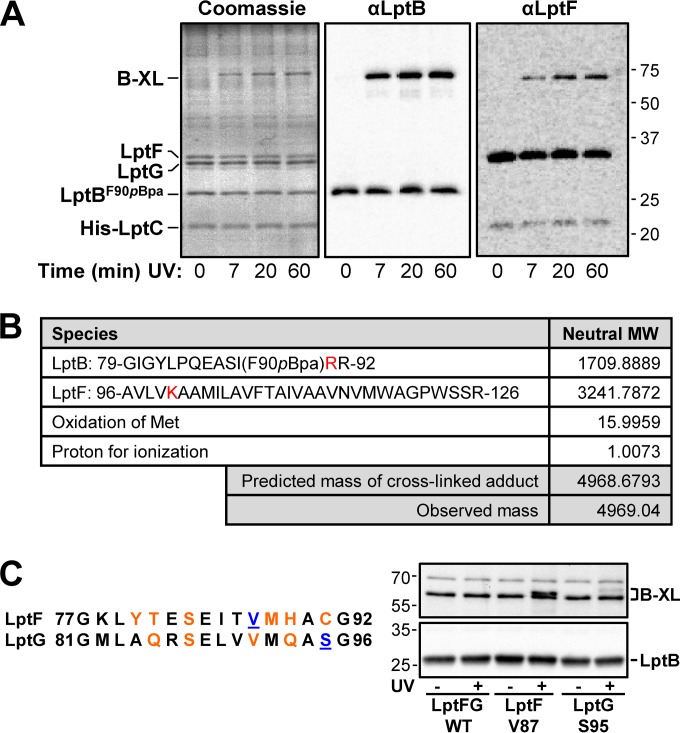
The functionally important cluster of residues in the LptB groove region directly contacts the coupling helices of LptFG. (A) Purified LptB^F90*p*BPA^-LptFG-LptC-His_7_ complexes were exposed to UV for the amounts of time indicated. A single LptB^F90pBPA^-LptF cross-link adduct (B-XL) was made visible by Coomassie blue staining and LptB and LptF immunoblot assays. The values to the right are molecular sizes in kilodaltons. (B) MS/MS analysis of peptide fragments of the LptB^F90pBPA^-LptFcross-link product reveals the appearance of a novel peptide (with the observed mass noted) that is not present in monomeric LptB and LptF (see Fig. S6 in the supplemental material). The predicted mass of the novel peptide (cross-linked adduct) is the sum of the species shown. Residues where trypsin failed to cleave the cross-linked adduct are red (see Fig. S6 for more details). MW, molecular weight. (C) Alignment of the amino acid sequences of the coupling helices of LptFG in *E. coli*. *p*Bpa substituted sites that cross-link to LptB are blue and underlined, while those that do not cross-link are orange (see Fig. S2 in the supplemental material). Immunoblot assay showing that LptF^V87pBPA^ and LptG^S95pBPA^ cross-link to LptB upon exposure to UV when produced from pBAD18LptFG3. LptFG WT is strain NR3720, which contains no *p*BPA substitutions. The values to the left are molecular sizes in kilodaltons.

If these conserved domains are indeed the coupling helices of LptFG, they should interact with the LptB groove. Because F90 in the groove of LptB interacts with LptFG, we examined if it makes contacts with (or near) the LptFG coupling helices by analyzing the *p*BPA cross-links formed by LptB^F90pBPA^ by tandem mass spectrometry (MS/MS). To obtain enough material for this analysis, we performed UV cross-linking experiments with purified LptB_2_FG-LptC-His_7_ complexes isolated from cells producing LptB^F90pBPA^. In this *in vitro* cross-linking system, we observed a single high-molecular-weight, UV-dependent band that increased in intensity over time (Fig. 2A, Coomassie blue-stained gel). This cross-link was detected with both LptB and LptF antisera (Fig. 2A). In this system, we cannot detect LptB^F90pBPA^-LptG cross-links. We separated the cross-linked complexes into individual components by denaturing electrophoresis and digested bands containing monomeric LptB and LptF and the LptB^F90pBPA^-LptF adduct with trypsin. We then analyzed the resulting peptides by liquid chromatography (LC)-MS/MS and searched for peptides that could be detected in the LptB^F90pBPA^-LptF sample but not in the LptB^F90pBPA^ and LptF samples. The *p*BPA-containing peptide encompassing residues 79 to 91 was detected in the monomeric LptB^F90pBPA^ sample (theoretical [m] = 1,553.7878, experimental *m*/*z* 1,554.892, see Fig. S6A and B in the supplemental material). We could not detect this peptide in the LptB^F90pBPA^-LptF adduct, as expected, because it was now covalently bound to its interacting LptF peptide (see Fig. S6B). We then searched for novel peaks of higher mass that were unique to the LptB^F90pBPA^-LptF cross-link and identified one with *m*/*z* 4,969.04 (see Fig. S6). This peptide corresponds to the adduct containing residues 79 to 92 of LptB^F90pBPA^ and 96 to 126 of LptF (Fig. 2B). The LptB^F90pBPA^ peptide would be generated if trypsin cleaved at R92 instead of the expected R91 because of steric hindrance caused by the cross-link at adjacent residue *p*BPA90. Likewise, the LptF peptide would be generated if trypsin failed to digest between residues 100 and 101 because of hindrance caused by a nearby cross-link. Thus, *p*BPA at position 90 in LptB likely cross-links with an LptF residue proximal to K100. Given that the predicted LptF coupling helix encompasses residues 77 to 92 and that the side chain of LptB residue 90 points upward from the edge of the groove, we propose that *p*BPA cross-links with a region that connects the coupling helix and transmembrane domain 3 in LptF.

To further support the idea that these domains are the coupling helices, we used *in vivo* cross-linking of functional LptFG variants containing *p*BPA within their coupling helices. After exposure of strains to UV, LptB immunoblot assays showed that LptF^V87pBPA^ and LptG^S95pBPA^ form cross-links with masses similar to those observed when *p*BPA was inserted into the LptB groove (Fig. 1C and 2C; see Fig. S3C and D). Taking these data together, we propose that these conserved domains indeed correspond to the LptFG coupling helices.

### The coupling helices of LptF and LptG play different roles in LPS transport.

**FIG 3:**
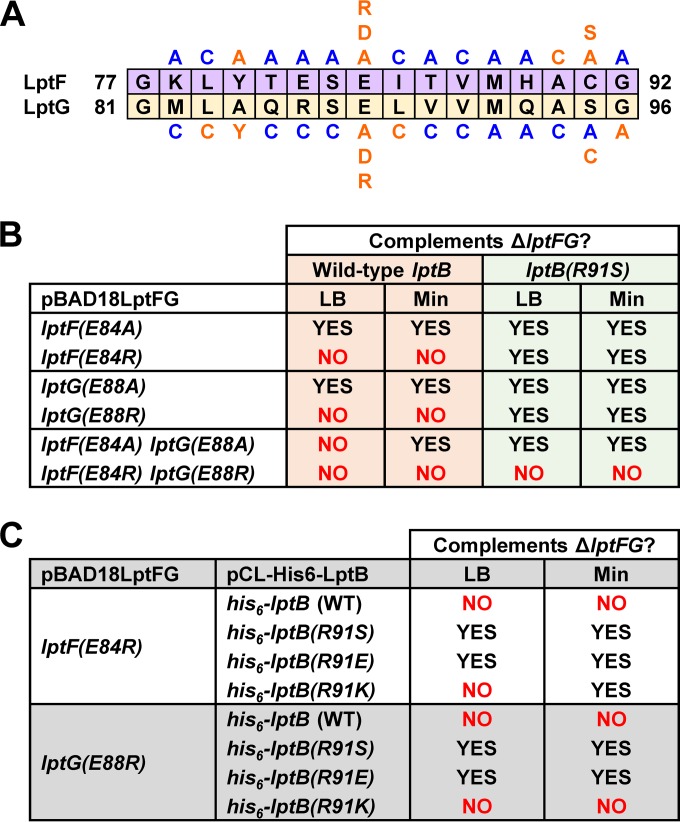
Defects in the coupling helices of LptFG are suppressed by changes adjacent to functionally important residues in the groove region of LptB. (A) Summary of functional analysis of the coupling helices of LptF and LptG. Substitutions (shown above the LptF sequence and below the LptG sequence) in pBAD18LptFG3 causing no detectable defects in strains carrying a chromosomal Δ*lptFG* allele are blue, while those resulting in defects are orange. (B) Abilities of various *lptF*(E84) and *lptG*(E88) alleles to complement a chromosomal Δ*lptFG* allele in LB and minimal medium (Min) in the presence of a chromosomal WT *lptB* or *lptB*(R91S) allele. (C) Abilities of various *lptB*(R91) alleles to suppress lethality caused by the *lptF*(E84R) and *lptG*(E88R) alleles.

Next, we tested whether residues within the coupling helices are important for LPS transport. We generated plasmid-encoded, coupling helix *lptFG* mutant alleles and tested if they could complement a chromosomal *lptFG* deletion in LB and minimal medium. The permeability of the OM of haploid mutants to hydrophobic antibiotics was monitored to assess the functional status of the Lpt system. We identified positions in the coupling helices that are important for Lpt function (Fig. 3A; see Fig. S7A). Defective LptFG variants were present at levels higher than those produced from chromosomal *lptFG*, indicating that the defects observed were not simply the result of reduced protein levels (see Fig. S7 in the supplemental material). We noticed that a pattern emerged for where functionally important residues are located within the coupling helices; residues in the center or at the ends of the coupling helices are critical for function, whereas many residues between them do not play important roles in LPS transport.

Residues that are important for LPS transport appear in a periodic fashion within the coupling helices (Fig. 3A). These residues are separated by approximately four or seven positions, suggesting that they are placed on the same face of each helix every one or two turns. For example, in LptF, we see defects with substitutions at positions 80, 84, 90, and 91. Likewise, in LptG, we see defects with substitutions at positions 83, 84, 88, 89, 95, and 96 (Fig. 3A). Consistent with this interpretation, these faces would also include the two positions (V87 in LptF and S95 in LptG) where *p*BPA substitutions cross-link to LptB (Fig. 2C).

Changes at equivalent positions within LptF and LptG do not always have the same effect on LPS function. For some positions, when the same substitution was made in LptF and LptG, the resulting variants were affected differently (Fig. 3A, compare LptF^L79C^ with LptG^L83C^, LptF^I85C^ with LptG^L89C^, LptF^A90C^ with LptG^A94C^, LptF^C91A^ with LptG^S95A^, and LptF^G92A^ with LptG^G96A^). Furthermore, swapping the identities of functionally important residues between the two helices also resulted in defects (Fig. 3A, compare LptF^Y80A^ with LptG^A84Y^ and LptF^C91S^ with LptG^S95C^). These results suggest that there might be functional differences between the coupling helices of LptFG. Given that these coupling helices contact the same ATPase, LptB, we propose that LptF and LptG might have distinct roles in coordinating the ATP hydrolysis cycle in the cytoplasm with LPS extraction at the periplasmic side of the IM.

### The conserved glutamate residues in the coupling helices of LptFG play a central role in LPS transport.

The most severe functional defects in the coupling helices were conferred by substitutions at the conserved glutamates. *lptF*(E84A) and *lptG*(E88A) single mutants were viable but exhibited OM permeability defects, while a *lptF*(E84A) *lptG*(E88A) double mutant was viable only in minimal medium (Fig. 3B; see Fig. S7). In addition, *lptF*(E84R) and *lptG*(E88R) single mutants were not viable (Fig. 3B). Thus, these conserved glutamates are crucial for LPS transport, possibly by mediating charge-based interactions. To test if the negative charge of the conserved glutamates is the only requirement for function, we replaced them with aspartate. The LptF^E84D^ and LptG^E88D^ variants were defective even though their protein levels were not affected (see Fig. S7 in the supplemental material). In fact, these aspartate variants were more defective than their alanine-substituted counterparts (see Fig. S7). These results suggest that the alanine and arginine substitutions eliminate important interactions mediated by the charge of the conserved glutamate residues. The aspartate substitutions do not restore this interaction and indeed cause additional defects that further compromise LptFG function.

### Defects in the LptFG coupling helices are suppressed by changing R91 next to the functional cluster of residues in the LptB groove.

To better understand how the conserved glutamates in the LptFG coupling helices affect LPS transport, we selected for suppressor mutations in the *lptF*(E84A) *lptG*(E88A) double mutant, which cannot grow on LB agar and is sensitive to hydrophobic antibiotics such as bacitracin in minimal medium (Fig. 3B; see Fig. S7). We characterized eight independent suppressors that could grow on LB plates containing bacitracin and found that all of them carried mutations in codon 91 of *lptB* changing residue R91 to cysteine, histidine, or serine. Because we could not detect any differences between the different suppressor alleles, we focused our studies on *lptB*(R91S). Notably, R91 is located in the groove of LptB adjacent to the functionally relevant cluster of residues, including F90 (Fig. 1).

To understand how changing R91 in LptB suppresses defects in the coupling helices of LptFG, we first investigated if suppression requires the loss of its positive charge. Changing R91 in LptB to serine or glutamate resulted in various degrees of suppression of *lptF* and *lptG* alleles that change the conserved coupling helix glutamate to either alanine or arginine (Fig. 3; Table 1). However, a lysine substitution at R91 could not suppress the lethality of the *lptG*(E88R) allele and only poorly restored growth to the *lptF*(E84R) mutant strain in minimal medium (Fig. 3C). These results suggest that suppression is achieved by the loss of an interaction mediated by the positive charge at position 91 of LptB.

**TABLE 1:** *lptB*(R91S) suppresses specific *lptFG* alleles, as determined by OM permeability defects in disc diffusion assays in LB

Strain	Relevant allele(s)	Diam (mm) of zone of inhibition^a^			
		Bac	Novo	Ery	Rif
NR754	*lptB^+^*	<6	<6	(8)	8
NR3602	*lptB*(R91S)	<6	<6	(8)	8
NR760	*lptD4213*	24 (25)	19 (24)	20	24
NR3601	*lptD4213*, *lptB*(R91S)	22 (24)	19 (23)	20	24
NR2761	*lptFG^+^*	<6	(8)	(13)	10 (11)
NR3265	*lptF*(E84A)	15	(8)	(18)	13 (20)
NR3590	*lptF*(E84A), *lptB*(R91S)	(9)	(7)	(15)	10 (11)
NR3680	*lptF*(E84D)	17 (22)	11 (13)	10 (24)	11 (24)
NR3703	*lptF*(E84D), *lptB*(R91S)	27	13	10 (29)	14 (27)
NR2762	*lptG*(E88A)	10	(14)	11 (23)	11 (17)
NR3592	*lptG*(E88A), *lptB*(R91S)	8	(10)	9 (14)	11 (12)
NR3681	*lptG*(E88D)	17 (19)	17	10 (27)	12 (27)
NR3704	*lptG*(E88D), *lptB*(R91S)	13	(10)	10 (18)	12 (18)

a No growth is denoted by values, and reduced growth is denoted by values in parentheses. No growth inhibition is denoted by a value of <6 (diameter of the disc). Bac, bacitracin; Novo, novobiocin; Ery, erythromycin; Rif, rifampin.

**FIG 4:**
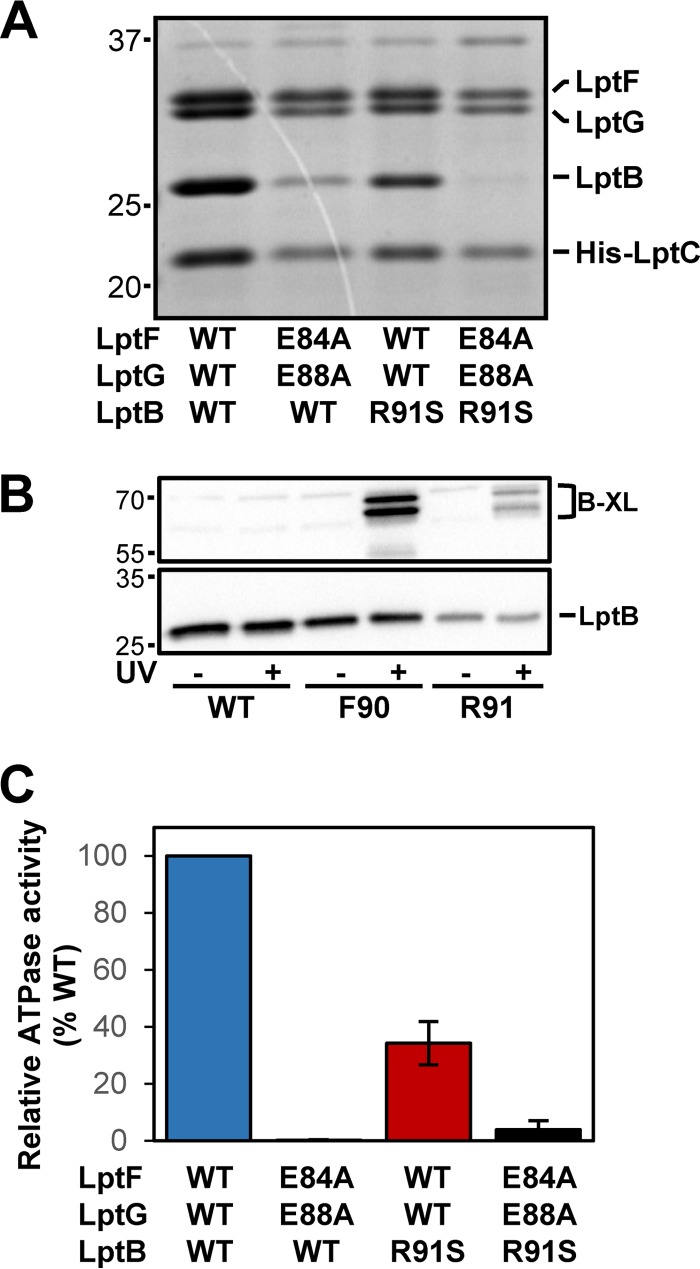
Residues in the groove region of LptB form a binding site for LptFG that controls the activity of the LptB_2_FG complex. (A) Coomassie blue-stained denaturing gel of purified LptB_2_FG-LptC-His_7_ complex variants. The values to the left are molecular sizes in kilodaltons. (B) LptB immunoblot assay of UV-dependent cross-links of His6-LptB^F90pBPA^ and His6-LptB^R91pBPA^ carried by pCL-His6-LptB in strains containing pBAD18LptFG3 and pSUP-BpaRS-6TRN. The WT is strain NR3720, which contains no *p*BPA substitutions in pCL-His6-LptB. The values to the left are molecular sizes in kilodaltons. (C) Relative ATPase activities of purified LptB_2_FG-LptC-His_7_ complexes determined by measuring the moles of inorganic phosphate released per mole of complex per minute with the WT complex as a reference.

We considered that defects conferred by replacing the negatively charged glutamates in the LptFG coupling helices could be simply caused by the loss of their interaction with positively charged R91 in LptB. If this hypothesis is correct, the *lptB*(R91S) single mutant and the *lptF*(E84A) *lptG*(E88A) double mutant should have similar phenotypes. However, several lines of evidence showed that this is not the case. In an otherwise WT strain, *lptB*(R91S) did not confer any OM permeability defects (Table 1). Furthermore, while purified LptB_2_FG-LptC-His_7_ complexes containing LptF^E84A^ LptG^E88A^ demonstrated weaker affinity for LptB^WT^ consistent with the defects observed *in vivo*, the compensatory charge change in LptB alone did not weaken the affinity of LptB^R91S^ for LptFG^WT^, nor did the presence of LptB^R91S^ restore the defective interaction with LptF^E84A^ LptG^E88A^ (Fig. 4A). Therefore, removal of charges at R91 in LptB and E84 in LptF and/or E88 in LptG does not result in the same defects. Elimination of the charge of R91 in LptB suppresses defects caused by changes in the coupling helix glutamates by altering an electrostatic interaction, improving LPS transport by modifying the activity of the Lpt system. We propose that this interaction is between R91 in LptB and an unidentified residue in LptF/G because R91 is positioned in the LptB groove adjacent to F90 and LptB^R91pBPA^ yields UV-dependent cross-links that are similar to those observed with LptB^F90pBPA^ (Fig. 4B).

### Substitutions of R91 in the groove region of LptB modify the activity of the LptB_2_FG complex.

Our results suggest that *lptB*(R91S) suppresses defects in LPS transport conferred by *lptF*(E84A/R) and *lptG*(E88A/R) alleles by altering the activity of the Lpt system because it can suppress OM permeability defects without restoring complex stability (Fig. 4A; Table 1). Since LPS transport depends on the ATPase activity of LptB, we tested if changing R91 in LptB alters the ATPase activity of purified LptB_2_FG-LptC-His_7_ complexes. Specifically, we compared the abilities of WT complexes and those containing LptF^E84A^ LptG^E88A^ and/or LptB^R91S^ to hydrolyze ATP. LptB^WT^-LptF^E84A^-LptG^E88A^ complexes had ca. 0.15% of the ATPase activity of WT complexes (Fig. 4C). This was not surprising, given the weaker affinity of LptF^E84A^ LptG^E88A^ for LptB (Fig. 4A). The R91S substitution in LptB caused a 25-fold increase in the ATPase activity of LptF^E84A^ LptG^E88A^ complexes even though the levels of LptB^R91S^ remained low (Fig. 4A and C). Surprisingly, the R91S substitution had the opposite effect on the ATPase activity of LptFG^WT^ complexes, reducing their activity by 65% (Fig. 4C). Thus, position 91 in LptB controls the activity of the LptB_2_FGC complex.

We suggest that R91 forms an interaction that affects LPS transport. This effect is different, depending on the status of the coupling helices of LptFG; losing this interaction reduces the ATPase activity of LptFG^WT^ complexes but increases that of LptF^E84A^ LptG^E88A^ complexes (Fig. 4C). These results imply that individually replacing the coupling helix glutamates in LptFG with alanines and losing the positive charge of R91 in LptB lower the ATPase activity of the LptB_2_FG complex by different mechanisms; however, their effects compensate each other when combined. Two predictions would follow if this interpretation is correct. First, *lptB*(R91S) should not be able to generally suppress other defects in Lpt components (i.e., is not a general suppressor of Lpt defects). Second, *lptB*(R91S) could improve or be detrimental to LPS transport when combined with mutations that differentially alter the function of the coupling helices (i.e., exhibit synthetic interactions).

We next addressed whether *lptB*(R91S) is a general suppressor of defects in LPS transport by combining it with different defective *lpt* alleles. We found that *lptB*(R91S) could not suppress the lethality of *lptB*(F90A) (groove defects) and *lptB*(H195A) (ATPase defects) (10). In addition, *lptB*(R91S) did not significantly change the OM permeability defects of an *lptD4213* (OM translocon defects) mutant (Table 1) (32). Thus, *lptB*(R91S) is not a general suppressor of Lpt defects.

Finally, to address the second prediction that changes at position 91 in LptB could differentially alter the activity of the transmembrane proteins LptFG, we introduced *lptB*(R91S) into strains carrying the *lptF*(E84D) and/or *lptG*(E88D) alleles. Earlier, we showed that changing the coupling helix glutamates to aspartates resulted in different (more severe) defects than those caused by the alanine substitutions. Here, we found that *lptB*(R91S) affects these *lptF* and *lptG* alleles differently. As described before, *lptB*(R91S) suppressed defects in both of the *lptF*(E84A) and *lptG*(E88A) single mutants (Fig. 3B; Table 1). We found that *lptB*(R91S) also partially suppressed OM permeability defects in the *lptG*(E88D) single mutant (Table 1). Surprisingly, *lptB*(R91S) worsened the OM permeability defects of the *lptF*(E84D) mutant (Table 1) and prevented it from growing in rich medium (i.e., conditional synthetic lethality). In agreement with this detrimental effect on *lptF*(E84D), *lptB*(R91S) was synthetic lethal with the *lptF*(E84D) *lptG*(E88D) allele pair. This stands in stark contrast to the observation that *lptB*(R91S) suppresses lethality in the *lptF*(E84A) *lptG*(E88A) double mutant (Fig. 3B). These results further support our earlier conclusion that replacing the conserved coupling helix glutamates with aspartate causes different defects than those caused by alanine or arginine substitutions. Furthermore, the fact that *lptB*(R91S) has different effects on equivalent *lptF* and *lptG* alleles is additional supporting evidence that the coupling helices of LptF and LptG have distinct roles in LPS transport. Taken together, these experiments implicate these three residues in the coordination of ATP hydrolysis by LptB with LptFG function to achieve LPS extraction.

## DISCUSSION

Organisms from all domains of life utilize ABC transporters to move cargo between compartments (33). The function of ABC transporters relies on the conversion of chemical energy (ATPase) into mechanical work, and a fundamental question that remains unanswered is how these machines couple ATP hydrolysis by their nucleotide-binding domains to substrate transport by their transmembrane partners. In Gram-negative bacteria, the LptB_2_FG transporter powers the transport of LPS across the envelope (8–11). How this transporter functions is poorly understood. In this study, we identify the coupling helices in LptF and LptG and characterize how these domains interact with the ATPase LptB. We demonstrate that there is a cluster of residues in LptB including F90, R91, L93, and R150 that forms a binding site that directly contacts the coupling helices of LptFG at one edge of the LptB groove, near the ATP-binding sites in the LptB dimer. We propose that residues R91 in the LptB groove and E84 and E88 in the coupling helices of LptF and LptG, respectively, coordinate ATP hydrolysis to achieve LPS extraction from the outer leaflet of the IM.

Defining the coupling helices of LptFG and residues within them that affect LPS extraction is critical to understanding how LptFG work with LptB. The coupling helix residues that we identified as affecting LPS transport are located on one face of the helices, presumably because they contact LptB. Some of these functional residues are located at the ends of the LptFG coupling helices and may be needed to properly position the two helices of LptFG within the LptB grooves. There are also conserved glutamates (E84 in LptF and E88 in LptG) that are crucial for the assembly of the LptB_2_FG complex since they mediate interactions that, when disrupted, lead to a decrease in the affinity of LptFG for LptB. The glutamates are strictly conserved, even among distant LptFG homologs, and also play a functional role in LPS release. Conservative substitutions of aspartate for these glutamates cause more severe defects than those resulting from alanine substitutions. We conclude that these substitutions create different problems for the LptB_2_FG ABC transporter and are not simply a result of differing degrees of the same defect because we obtained opposite phenotypes (i.e., suppression versus lethality) when combining a single change in the LptB groove (R91S) with either alanine or aspartate substitutions at the conserved glutamates. Thus, the glutamates in the coupling helices are critical for both the assembly and function of the LptB_2_FG complex.

In other ABC transporters, an EAA motif with conserved glutamates in the coupling helices is also important for both the assembly and the function of the transporter (30, 31, 34). It has been proposed that conformational changes in the EAA motifs occur during the ATPase cycle (34). We propose that the proper placement of the ends of the coupling helices within the grooves of LptB is required to correctly position the crucial glutamate residues in the LptFG coupling helices throughout the ATPase cycle so that the activity of LptB can be coordinated with LPS extraction by LptFG. In addition, we propose that even though the coupling helices of LptF and LptG interact with the same ATPase LptB, they do not function symmetrically. We reached this conclusion because swapping important residues between the two helices results in defects. Also supporting this conclusion is the fact that identical substitutions at equivalent positions in LptF and LptG result in different phenotypes, similar to what was reported for the coupling helices of MalFG in the maltose transporter, which serves as a prototype for ABC importers (30, 34). That LptB might not symmetrically transduce movement to LptFG during the ATPase cycle suggests that LptF and LptG perform different roles in the extraction of LPS from the IM.

The cluster of residues we identified in LptB, which is located at the edge of the groove that is proximal to the LptB dimer interface, forms an important binding site for the coupling helices to monitor the ATP hydrolysis cycle. Three residues in this cluster, F90, R91, and L93, are located within the Q loop of LptB just above the signature motif (10). Both of these motifs are conserved in ABC transporters and are thought to be important for coupling of ATP hydrolysis with the function of their transmembrane domains (24). The Q loop includes the defining glutamine (Q85 in LptB) that coordinates the Mg^2+^ ion that is required for proper positioning of ATP and catalysis (10, 24). We have shown that residues within this Q loop interact directly with LptFG and imagine that as ATP is converted to ADP, structural changes in the Q loop could be directly communicated to LptFG. We previously obtained structures of LptB in both an ATP- and an ADP-bound state and noted that one of the largest structural changes when comparing pre- and post-ATP hydrolysis occurs within the Q loop (10). During hydrolysis, Q-loop residues, including F90 and R91, move down and away from their original position. Therefore, concomitant movement of the LptFG coupling helices would be required to maintain contact with positions 90 and 91 in LptB after ATP hydrolysis. This movement could be necessary for function of the complex to facilitate LPS extraction. Indeed, connection of the coupling helices, the Q loop, and the signature motif has also been described in the MalK_2_FG system. Substitutions in the Q loop and near the signature motif of the ATPase MalK suppress defects in the EAA motif of the coupling helices of MalFG (30), and interactions between the EAA domains and Q loop are modulated by ATP, inducing conformational changes in the EAA motifs that are crucial for function (34, 35).

How are R91 in LptB and the glutamates in the coupling helices of LptFG involved in the coupling of ATPase activity and LPS extraction? Individually, changes to these residues have a negative effect on the ATPase activity and assembly of LptB with LptFG. However, when changes to these residues are present in the same complex, they appear to offset each other, resulting in suppression. We have proposed that R91 mediates an ionic interaction with LptFG that, when lost, alters the ATPase activity of LptB. Depending on the functional status of the coupling helices, substitutions at R91 in LptB result in either a decrease in ATPase activity (when the coupling helices are fully functional) or an increase in ATPase activity (when the conserved glutamates of the LptFG coupling helices are replaced with alanines). These opposing effects on ATPase activity suggest that these residues affect different steps in the ATPase cycle of the LptB_2_FG complex.

Many Gram-negative bacteria can be naturally resistant to antibiotics because their cell surface is covered with LPS, which efficiently prevents the entry of many hydrophobic antimicrobials (3). The discovery of sites in the LptB_2_FG complex that modulate its activity might lead to the development of small molecules that could interfere with LPS transport, leading to loss of the impermeability of these bacteria to antimicrobials. In addition, the approaches and findings described here might be applicable to a related Lol ABC transporter that extracts lipoproteins from the IM of Gram-negative bacteria (1).

## MATERIALS AND METHODS

### Strains and growth conditions.

For the strains used in this study, see Table S1 in the supplemental material. For details of strain construction and growth conditions, see Text S1 in the supplemental material.

### Genetic characterization of mutant alleles.

The functionality of *lpt* alleles was assessed by using a system based on plasmids derived from partitioning-defective plasmid pRC7 (10, 36). pRC7-derived plasmids, carrying *lacZ* and either WT *lptB* or *lptFG*, are readily lost in the absence of antibiotic selection unless they carry the only functional copy of the essential *lpt* gene in a particular strain. The LptB characterization strain NR2050 was previously described (10). Construction of the analogous LptFG characterization strain NR2759 is described in Text S1 in the supplemental material. Mutant *lpt* alleles carried by either the pET23/42LptB or the pBAD18LptFG3 plasmid were introduced into NR2050 or NR2759, respectively, and transformants were selected on LB containing ampicillin, isopropyl-β-d-thiogalactopyranoside (IPTG), and 5-bromo-4-chloro-3-indolyl-β-d-galactopyranoside (X-Gal). If a mutant allele could sustain cell viability, the resulting strain lost the pRC7-derived plasmid and produced white colonies (LacZ^−^), whereas loss-of-function alleles yielded blue colonies (LacZ^+^) that required the maintenance of the pRC7-derived plasmid. Functionality was checked on both LB and glucose minimal medium.

### OM permeability assays.

We assessed the OM permeability of strains to hydrophobic antibiotics (bacitracin, novobiocin, erythromycin, and rifampin) by disc diffusion assays (10). The data shown are representative of at least three independent experiments.

### Immunoblotting.

Samples from overnight cultures were normalized for cell density and subjected to 10% SDS-polyacrylamide gel electrophoresis (10). Proteins were transferred to polyvinylidene difluoride membranes (Roche Diagnostics), which were probed with anti-LptB (1:10,000 dilution), anti-LptF (1:10,000 dilution; gift from the Polissi laboratory), or anti-LptG (1:5,000 dilution) antiserum, followed by horseradish peroxidase-conjugated anti-rabbit antibodies (1:10,000 dilution; GE, Amersham). A signal was developed with Clarity Western ECL substrate (Bio-Rad) and imaged with a ChemiDoc XRS+ system and ImageLab 5.2.1 software (Bio-Rad).

### *In vivo* photo-cross-linking.

Amber codons were introduced by site-directed mutagenesis into pET23/42LptB, pET23/42His6-LptB, pCL-His6-LptB, or pBAD18LptFG3 (see Table S1 and S2 in the supplemental material) as described in Text S1 in the supplemental material. Overnight cultures were diluted in 5 to 6 ml of glucose minimal medium containing *p*BPA and the appropriate antibiotics to an optical density at 600 nm (OD_600_) of 0.1 to 0.2. Strains with *p*BPA-containing *lptB* alleles were then grown to an OD_600_ of 0.6 to 0.8, while those with *p*BPA-containing *lptFG* alleles were grown to an OD_600_ of 1.0 to 1.2. In a 24-well flat-bottom cell culture plate (Costar; Corning Inc.), 2-ml volumes of cultures were irradiated for 10 min with a UV lamp (365 nm; Spectroline E series). An additional 1 ml of culture was set aside as a UV-untreated control. Samples were analyzed by immunoblotting.

### *In vitro* photo-cross-linking of purified LptBFGC-His complexes and trypsin digestion for MS.

The method used to purify LptB^F90*p*BPA^FG-LptC-His_7_ complexes is modified from that of Sherman et al. (10) and is described in Text S1 in the supplemental material. To produce cross-linked complexes, an LptB^F90*p*BPA^FG-LptC-His protein solution was UV irradiated at 365 nm on ice for 1 h and then either frozen at −80°C or immediately mixed with 2× SDS loading dye (100 mM Tris-HCl [pH 6.8], 4% [wt/vol] SDS, 0.05% [wt/vol] bromothymol blue, 20% [vol/vol] glycerol, 5% [vol/vol] β-mercaptoethanol), boiled for 10 min, loaded onto a 14% SDS-polyacrylamide gel, and visualized with Coomassie blue stain (adapted from reference 37). We identified bands by immunoblotting. Coomassie blue-stained bands corresponding to LptB^F90pBPA^, LptF, LptG, and LptB^F90pBPA^-LptF were excised from the gel and prepared for matrix-assisted laser desorption ionization–time of flight (MALDI-TOF) and LC-MS analyses of tryptic peptides as described in Text S1 in the supplemental material.

### Expression and purification of LptB_2_FGC complexes for ATPase assay.

Strains NR2761 [Δ*lptFG*::*frt*(pBAD18LptFG3)] and NR3327 [Δ*lptFG*::*frt*(pBAD18LptFG3/LptFE84A/LptGE88A)] transformed with pCDFduet-LptB-LptC-His7 or pCDFduet-LptB(R91S)-LptC-His7 were used to purify LptB_2_FGC complexes and measure their ATPase activities as described Text S1 in the supplemental material.

## SUPPLEMENTAL MATERIAL

Figure S1  Structure-function analysis of LptB groove variants. (A) OM permeability defects of haploid strains carrying mutant *lptB* alleles in pET23/42LptB were assessed by disc diffusion assay with four antibiotics. Shown is a representative data set for LptB variants that have increased sensitivity (≥3 mm) to at least one antibiotic. Variants not listed reproducibly had no increased sensitivity (≤3 mm) with respect to strain NR2101, which expresses the WT *lptB^+^* allele from pET23/42LptB. The data shown are representative of at least three independent experiments. The diameters (in millimeters) of zones with no growth are shown as values, and those of zones with reduced growth are shown as values in parentheses; no visible zone of inhibition is shown as a value less than the diameter of the disc (6 mm). (B) Immunoblot assay showing levels of defective variant LptB proteins in cultures grown overnight in rich (LB) or minimal (M63gluc) medium. The designation 754 refers to NR754, the WT strain expressing *lptB* from its native chromosomal locus (M. J. Casadaban, J Mol Biol **104:**541–555, 1976; N. Ruiz, L. S. Gronenberg, D. Kahne, and T. J. Silhavy, Proc Natl Acad Sci U S A **105:**5537–5542, 2008). For haploid *lptB* strains, the WT is strain NR2101, which carries pET23/42LptB^WT^. For merodiploid *lptB* strains, the WT is strain NR2583, which produced LptB^WT^ from both chromosomal *lptB* and pET23/42LptB. As described in Materials and Methods, samples were normalized by the OD_600_ of their cultures. Download Figure S1, PDF file, 0.3 MB

Figure S2  Conservation of LptB residues shown in a ClustalW (M. A. Larkin, G. Blackshields, N. P. Brown, R. Chenna, P. A. McGettigan, H. McWilliam, F. Valentin, I. M. Wallace, A. Wilm, R. Lopez, J. D. Thompson, T. J. Gibson, and D. G. Higgins, Bioinformatics **23:**2947–2948, 2007; J. D. Thompson, D. G. Higgins, and T. J. Gibson, Nucleic Acids Res **22:**4673–4680, 1994) alignment of LptB homologs with groove-exposed residues investigated in this study underlined with red bars. Residues F90, L93, and R150 in *E. coli* LptB are marked with yellow asterisks. Coloring was done in Jalview (A. M. Waterhouse, J. B. Procter, D. M. Martin, M. Clamp, and G. J. Barton, Bioinformatics **25:**1189–1191, 2009) with the following percentage identity color scheme: light purple, 50% identical; dark purple, 100% identical; white, <50% identical. Homologs were identified from *Caulobacter crescentus* NA1000 (*Alphaproteobacteria*), *Neisseria meningitidis* Fam18 (*Betaproteobacteria*), *Escherichia coli* K-12 strain MG1655 (*Gammaproteobacteria*), *Geobacter uraniireducens* Rfr (*Deltaproteobacteria*), *Campylobacter jejuni* RM1221 (*Epsilonproteobacteria*), *Gramella forsetii* KT0803 (*Bacteroidetes*), *Elusimicrobium minutum* Pei191 (*Elusimicrobia*), *Sulfurihydrogenibium azorense* Az-Fu1 (*Aquificae*), *Denitrovibrio acetiphilus* DSM 12809 (*Deferribacteres*), and *Anabaena variabilis* ATCC 29413 (*Cyanobacteria*). Download Figure S2, PDF file, 0.6 MB

Figure S3  *p*Bpa-containing LptB and LptFG variants that do not interact directly with partners. (A) Heightened exposure and contrast of the LptB immunoblot assay in Fig. 1C showing low-efficiency cross-links from R92*p*Bpa and Q104*p*Bpa (top, exposure for 250 s and high contrast [20,000]; middle, exposure for 150 s and high contrast [52,000]). (B) Immunoblot assay showing LptB variants containing *p*BPA substitutions that do not yield detectable UV-dependent cross-links. The WT is strain NR3877, which contains no *p*BPA substitutions in pET23/42LptB. (C, D) Immunoblot assay of LptF-*p*BPA (C) and LptG-*p*BPA (D) variants that do not yield detectable UV-dependent cross-links to LptB. The WT is strain NR3720, which contains no *p*BPA substitutions in pBAD18LptFG3. Download Figure S3, PDF file, 1.6 MB

Figure S4  LptB^F90pBpa^ cross-links to LptFGB. Shown are LptB (left), LptF (center), and LptG (right) immunoblot assays of strain NR3540 carrying pCL-His6-LptB^F90pBPA^ and pBAD18LptFG3 to increase levels of LptFG. The WT is strain NR3720, which contains no *p*BPA substitutions in pCL-His6-LptB. The LptB^F90pBpa^-LptF and LptG cross-links are recognized by antisera raised against LptB, LptF, and LptG and designated B-XL, F-XL, and G-XL, respectively. Download Figure S4, PDF file, 0.6 MB

Figure S5  Alignment of distant LptF and LptG homologs. ClustalW (M. A. Larkin, G. Blackshields, N. P. Brown, R. Chenna, P. A. McGettigan, H. McWilliam, F. Valentin, I. M. Wallace, A. Wilm, R. Lopez, J. D. Thompson, T. J. Gibson, and D. G. Higgins, Bioinformatics **23:**2947–2948, 2007; J. D. Thompson, D. G. Higgins, and T. J. Gibson, Nucleic Acids Res **22:**4673–4680, 1994) alignment shows six conserved, highly hydrophobic regions that correlate with transmembrane domains of *E. coli* LptF (marked with orange lines) and LptG (marked with purple lines) predicted by TMSEG (Predictprotein) (G. Yachdav G, Kloppmann E, L. Kajan, M. Hecht, T. Goldberg, T. Hamp, P. Honigschmid, A. Schafferhans, M. Roos, M. Bernhofer, L. Richter, H. Ashkenazy, M. Punta, A. Schlessinger, Y. Bromberg, Schneider R, G. Vriend, C. Sander, N. Ben-Tal, and B. Rost, Nucleic Acids Res **42:**W337–W343, 2014). Homologs from *Caulobacter crescentus* NA1000 (*Alphaproteobacteria*), *Neisseria meningitidis* Fam18 (*Betaproteobacteria*), *Escherichia coli* K-12 strain MG1655 (*Gammaproteobacteria*), *Geobacter uraniireducens* Rfr (*Deltaproteobacteria*), *Campylobacter jejuni* RM1221 (*Epsilonproteobacteria*), *Gramella forsetii* KT0803 (*Bacteroidetes*), *Elusimicrobium minutum* Pei191 (*Elusimicrobia*), *Sulfurihydrogenibium azorense* Az-Fu1 (*Aquificae*), *Denitrovibrio acetiphilus* DSM 12809 (*Deferribacteres*), and *Anabaena variabilis* ATCC 29413 (*Cyanobacteria*) were identified. Coloring was done in Jalview (A. M. Waterhouse, J. B. Procter, D. M. Martin, M. Clamp, and G. J. Barton, Bioinformatics **25:**1189–1191, 2009) with the following ClustalX color scheme: light blue, hydrophobic; green, hydrophilic; orange, Gly; yellow, Pro; magenta, negatively charged; red, positively charged. Download Figure S5, PDF file, 1.2 MB

Figure S6  Identification of cross-link-containing peptides in LptB^F90pBPA^-LptF adducts via MALDI-TOF and LC-MS/MS. (A) Table showing tryptic digest peptides of LptB^F90pBPA^ and LptF that are part of the cross-linked peptide described in Fig. 2, listed with the monoisotopic mass of each neutral peptide or modification. Residues where trypsin failed to cleave the cross-link adduct are red. The predicted mass of the cross-linked adduct is the sum of the species highlighted in blue. (B) MALDI-TOF traces for trypsin-digest LptB^F90pBPA^ (bottom) and LptB^F90pBPA^-LptF adduct (top) in the *m*/*z* range of 1,550 to 1,560. From trypsin-digested LptB^F90pBPA^, we expected to see peptide 79-GIGYLPQEASI(F90pBPA)R-91 at [M+H] = 1,554.8919. As expected, this was observed in the un-cross-linked sample but not in the cross-linked sample, presumably because *p*BPA has cross-linked something. The overlapping peak at *m*/*z* 1,552.882 could not be identified and is likely a contaminant; notably, its abundance is roughly equal between the two samples, whereas the abundance of the *m*/*z* 1,554.8919 peak decreases greatly. (C) The LptF peptide 96-AVLVK-100 is adjacent to the coupling helix and has a theoretical [M+H] of 529.3708. The two panels show intensity versus time at *m*/*z* 529.3708 ± 0.005 for the LptF (top) and LptB^F90pBPA^-LptF adduct (bottom) samples run on LC-MS. (D) Masses detected in the range of 6.7 to 7.2 min in the LptF sample. The high peak at 529.3717 is within the error of the detector for the correct mass for the LptF peptide AVLVK. (E) To detect larger, potentially cross-linked peptides, low-resolution analysis of digested LptB^F90pBPA^, LptF, LptG, and LptB^F90pBPA^-LptF adduct was performed by MALDI-TOF in linear mode with the *m*/*z* range expanded. A unique peak is present in the LptB^F90pBPA^-LptF sample, which we hypothesize is an adduct of LptB^F90pBPA^(79-92) with LptF(96-126). Download Figure S6, PDF file, 0.3 MB

Figure S7  Structure-function analysis of LptFG coupling helix variants. (A) Permeability of OM of haploid *lptFG* mutants measured by disc diffusion assay with four antibiotics as described in Fig. S1A. Strains carry *lptFG* alleles on pBAD18LptFG3 derivatives. NR2761 was used as the WT *lptFG^+^* control. The sequences of the LptFG coupling helices are shown at the top. (B) Levels of defective variant LptFG proteins in cultures grown overnight. LptF and LptG immunoblot assays of haploid strains for partial loss-of-function LptF (left) and LptG (right) variants, respectively, grown in LB. (C) LptF and LptG immunoblot assays of merodiploid strains for total loss-of-function variants grown in LB and of haploid strains for conditional loss-of-function variants grown in glucose minimal medium. The designation 754 refers to strain NR754, which was used as the control for chromosomally produced LptFG. For haploid strains, the WT is NR2761. For merodiploid strains, the WT is NR3079. Download Figure S7, PDF file, 0.7 MB

Table S1  Strains used in this study. Table S1, DOCX file, 0.02 MB

Table S2  Primers used in this study. Primers used for the blunt-end site-directed mutagenesis with ligation protocol are marked with asterisks. Table S2, DOCX file, 0.02 MB

Text S1  Supplemental materials and methods used in this study. Download Text S1, DOCX file, 0.1 MB
