# Supplementary material for: Identification of Residues in the Lipopolysaccharide ABC Transporter That Coordinate ATPase Activity with Extractor Function
Source: mBio. 2016 Oct 18;7(5):e01729-16. doi: 10.1128/mBio.01729-16 (PMC5082905; doi:10.1128/mBio.01729-16)
Supplement: Figure S1 — Structure-function analysis of LptB groove variants. (A) OM permeability defects of haploid strains carrying mutant lptB alleles in pET23/42LptB were assessed by disc diffusion assay with four antibiotics. Shown is a representative data set for LptB variants that have increased sensitivity (≥3 mm) to at least one antibiotic. Variants not listed reproducibly had no increased sensitivity (≤3 mm) with respect to strain NR2101, which expresses the WT lptB+ allele from pET23/42LptB. The data shown are representative of at least three independent experiments. The diameters (in millimeters) of zones with no growth are shown as values, and those of zones with reduced growth are shown as values in parentheses; no visible zone of inhibition is shown as a value less than the diameter of the disc (6 mm). (B) Immunoblot assay showing levels of defective variant LptB proteins in cultures grown overnight in rich (LB) or minimal (M63gluc) medium. The designation 754 refers to NR754, the WT strain expressing lptB from its native chromosomal locus (M. J. Casadaban, J Mol Biol 104:541–555, 1976; N. Ruiz, L. S. Gronenberg, D. Kahne, and T. J. Silhavy, Proc Natl Acad Sci U S A 105:5537–5542, 2008). For haploid lptB strains, the WT is strain NR2101, which carries pET23/42LptBWT. For merodiploid lptB strains, the WT is strain NR2583, which produced LptBWT from both chromosomal lptB and pET23/42LptB. As described in Materials and Methods, samples were normalized by the OD600 of their cultures. Download [file mbo005163035sf1.pdf]

A

		Zone of inhibition (in mm)			
**Relevant allele**	**Media**	**Bacitracin**	**Novobiocin**	**Erythromycin**	**Rifampicin**
***lptB*+**	**LB**	<6	<6	(8)	8 (9)
lptB(R150A)	**LB**	10	(10)	11 (17)	10 (11)
***lptB*+**	**M63gluc**	<6	(14)	(13)	8 (9)
lptB(F90I)	**M63gluc**	19	(19)	(34)	35
lptB(L93F)	**M63gluc**	13	(14)	(24)	22 (30)

B

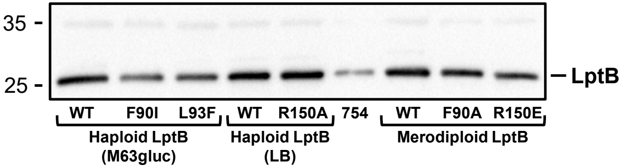

FIG S1
