# Supplementary figures and images for: Identification of Residues in the Lipopolysaccharide ABC Transporter That Coordinate ATPase Activity with Extractor Function
Source: mBio. 2016 Oct 18;7(5):e01729-16. doi: 10.1128/mBio.01729-16 (PMC5082905; doi:10.1128/mBio.01729-16)

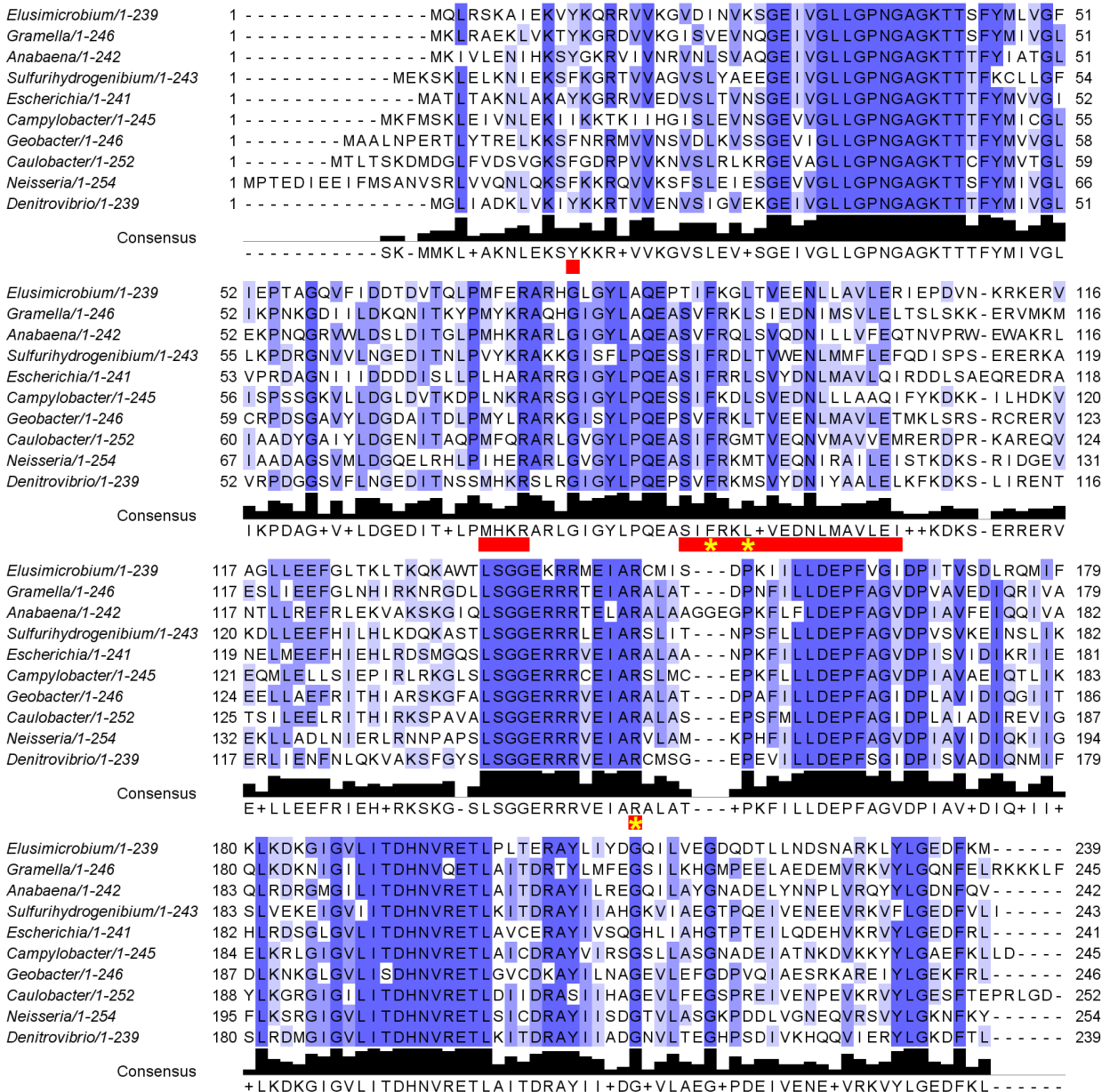

FIG S2

Supplement: Figure S2 — Conservation of LptB residues shown in a ClustalW (M. A. Larkin, G. Blackshields, N. P. Brown, R. Chenna, P. A. McGettigan, H. McWilliam, F. Valentin, I. M. Wallace, A. Wilm, R. Lopez, J. D. Thompson, T. J. Gibson, and D. G. Higgins, Bioinformatics 23:2947–2948, 2007; J. D. Thompson, D. G. Higgins, and T. J. Gibson, Nucleic Acids Res 22:4673–4680, 1994) alignment of LptB homologs with groove-exposed residues investigated in this study underlined with red bars. Residues F90, L93, and R150 in E. coli LptB are marked with yellow asterisks. Coloring was done in Jalview (A. M. Waterhouse, J. B. Procter, D. M. Martin, M. Clamp, and G. J. Barton, Bioinformatics 25:1189–1191, 2009) with the following percentage identity color scheme: light purple, 50% identical; dark purple, 100% identical; white, <50% identical. Homologs were identified from Caulobacter crescentus NA1000 (Alphaproteobacteria), Neisseria meningitidis Fam18 (Betaproteobacteria), Escherichia coli K-12 strain MG1655 (Gammaproteobacteria), Geobacter uraniireducens Rfr (Deltaproteobacteria), Campylobacter jejuni RM1221 (Epsilonproteobacteria), Gramella forsetii KT0803 (Bacteroidetes), Elusimicrobium minutum Pei191 (Elusimicrobia), Sulfurihydrogenibium azorense Az-Fu1 (Aquificae), Denitrovibrio acetiphilus DSM 12809 (Deferribacteres), and Anabaena variabilis ATCC 29413 (Cyanobacteria). Download [file mbo005163035sf2.pdf]

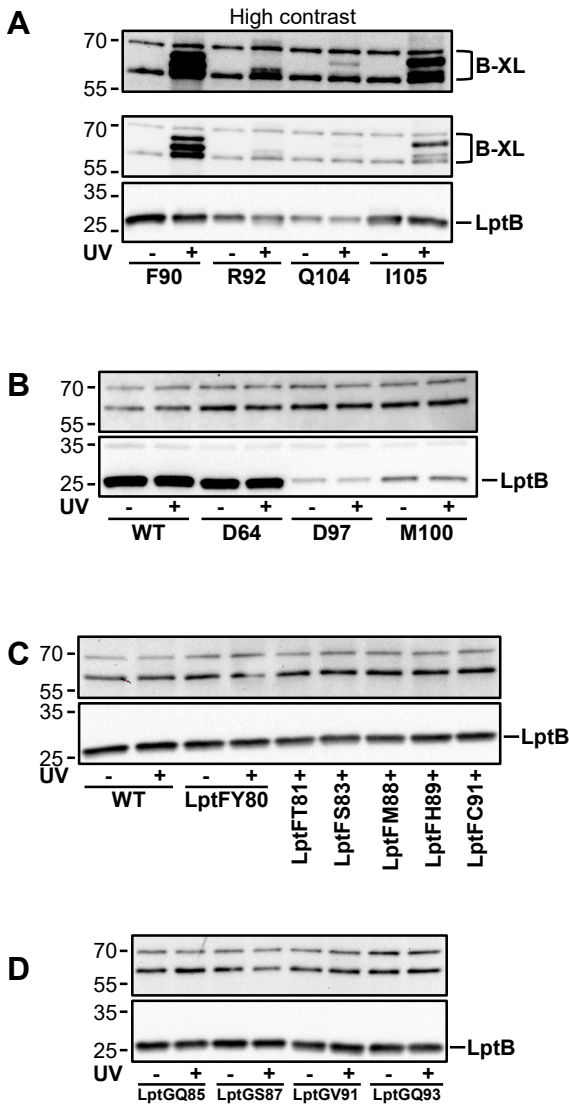

**FIG S3**

Supplement: Figure S3 — pBpa-containing LptB and LptFG variants that do not interact directly with partners. (A) Heightened exposure and contrast of the LptB immunoblot assay in Fig. 1C showing low-efficiency cross-links from R92pBpa and Q104pBpa (top, exposure for 250 s and high contrast [20,000]; middle, exposure for 150 s and high contrast [52,000]). (B) Immunoblot assay showing LptB variants containing pBPA substitutions that do not yield detectable UV-dependent cross-links. The WT is strain NR3877, which contains no pBPA substitutions in pET23/42LptB. (C, D) Immunoblot assay of LptF-pBPA (C) and LptG-pBPA (D) variants that do not yield detectable UV-dependent cross-links to LptB. The WT is strain NR3720, which contains no pBPA substitutions in pBAD18LptFG3. Download [file mbo005163035sf3.pdf]

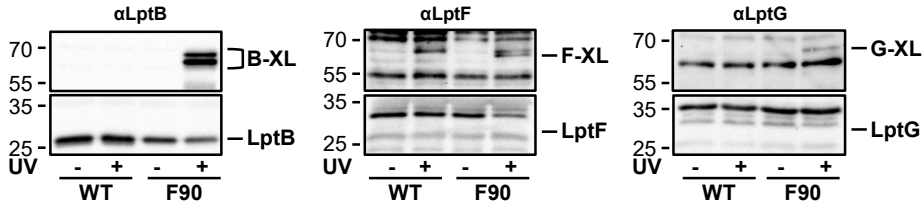

**FIG S4**

Supplement: Figure S4 — LptBF90pBpa cross-links to LptFGB. Shown are LptB (left), LptF (center), and LptG (right) immunoblot assays of strain NR3540 carrying pCL-His6-LptBF90pBPA and pBAD18LptFG3 to increase levels of LptFG. The WT is strain NR3720, which contains no pBPA substitutions in pCL-His6-LptB. The LptBF90pBpa-LptF and LptG cross-links are recognized by antisera raised against LptB, LptF, and LptG and designated B-XL, F-XL, and G-XL, respectively. Download [file mbo005163035sf4.pdf]
