# Supplementary material for: Identification of Residues in the Lipopolysaccharide ABC Transporter That Coordinate ATPase Activity with Extractor Function
Source: mBio. 2016 Oct 18;7(5):e01729-16. doi: 10.1128/mBio.01729-16 (PMC5082905; doi:10.1128/mBio.01729-16)
Supplement: Figure S6 — Identification of cross-link-containing peptides in LptBF90pBPA-LptF adducts via MALDI-TOF and LC-MS/MS. (A) Table showing tryptic digest peptides of LptBF90pBPA and LptF that are part of the cross-linked peptide described in Fig. 2, listed with the monoisotopic mass of each neutral peptide or modification. Residues where trypsin failed to cleave the cross-link adduct are red. The predicted mass of the cross-linked adduct is the sum of the species highlighted in blue. (B) MALDI-TOF traces for trypsin-digest LptBF90pBPA (bottom) and LptBF90pBPA-LptF adduct (top) in the m/z range of 1,550 to 1,560. From trypsin-digested LptBF90pBPA, we expected to see peptide 79-GIGYLPQEASI(F90pBPA)R-91 at [M+H] = 1,554.8919. As expected, this was observed in the un-cross-linked sample but not in the cross-linked sample, presumably because pBPA has cross-linked something. The overlapping peak at m/z 1,552.882 could not be identified and is likely a contaminant; notably, its abundance is roughly equal between the two samples, whereas the abundance of the m/z 1,554.8919 peak decreases greatly. (C) The LptF peptide 96-AVLVK-100 is adjacent to the coupling helix and has a theoretical [M+H] of 529.3708. The two panels show intensity versus time at m/z 529.3708 ± 0.005 for the LptF (top) and LptBF90pBPA-LptF adduct (bottom) samples run on LC-MS. (D) Masses detected in the range of 6.7 to 7.2 min in the LptF sample. The high peak at 529.3717 is within the error of the detector for the correct mass for the LptF peptide AVLVK. (E) To detect larger, potentially cross-linked peptides, low-resolution analysis of digested LptBF90pBPA, LptF, LptG, and LptBF90pBPA-LptF adduct was performed by MALDI-TOF in linear mode with the m/z range expanded. A unique peak is present in the LptBF90pBPA-LptF sample, which we hypothesize is an adduct of LptBF90pBPA(79-92) with LptF(96-126). Download [file mbo005163035sf6.pdf]

A

Species	Neutral MW
LptB: 79-GIGYLPQEASI(F90*p*BPA)R-91	1553.7878
LptB: 79-GIGYLPQEASI(F90*p*BPA)RR-92	1709.8889
LptF: 96-AVLVK	528.3635
LptF: 101-AAMILAVFTAIVAAVNVMWAGPWSSR-126	2731.4342
LptF: 96-AVLVKAAMILAVFTAIVAAVNVMWAGPWSSR-126	3241.7872
Oxidation of Met	15.9959
Proton for ionization	1.0073
Predicted mass of cross-linked adduct	4968.6793
Observed mass	4969.04

B

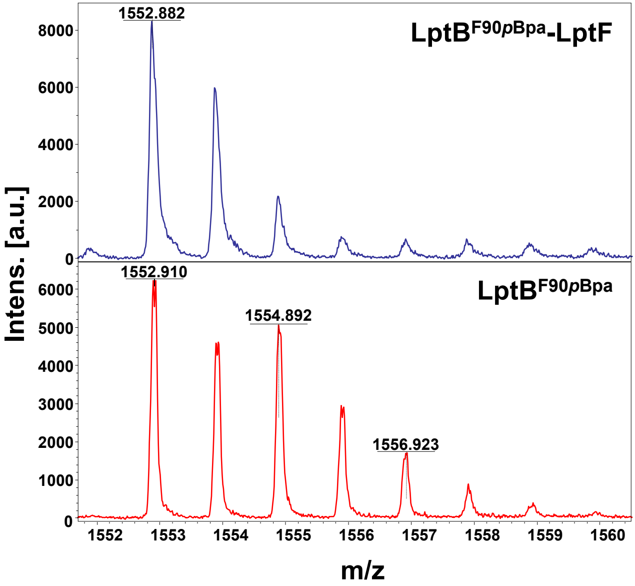

FIGS6

Fig. S6.

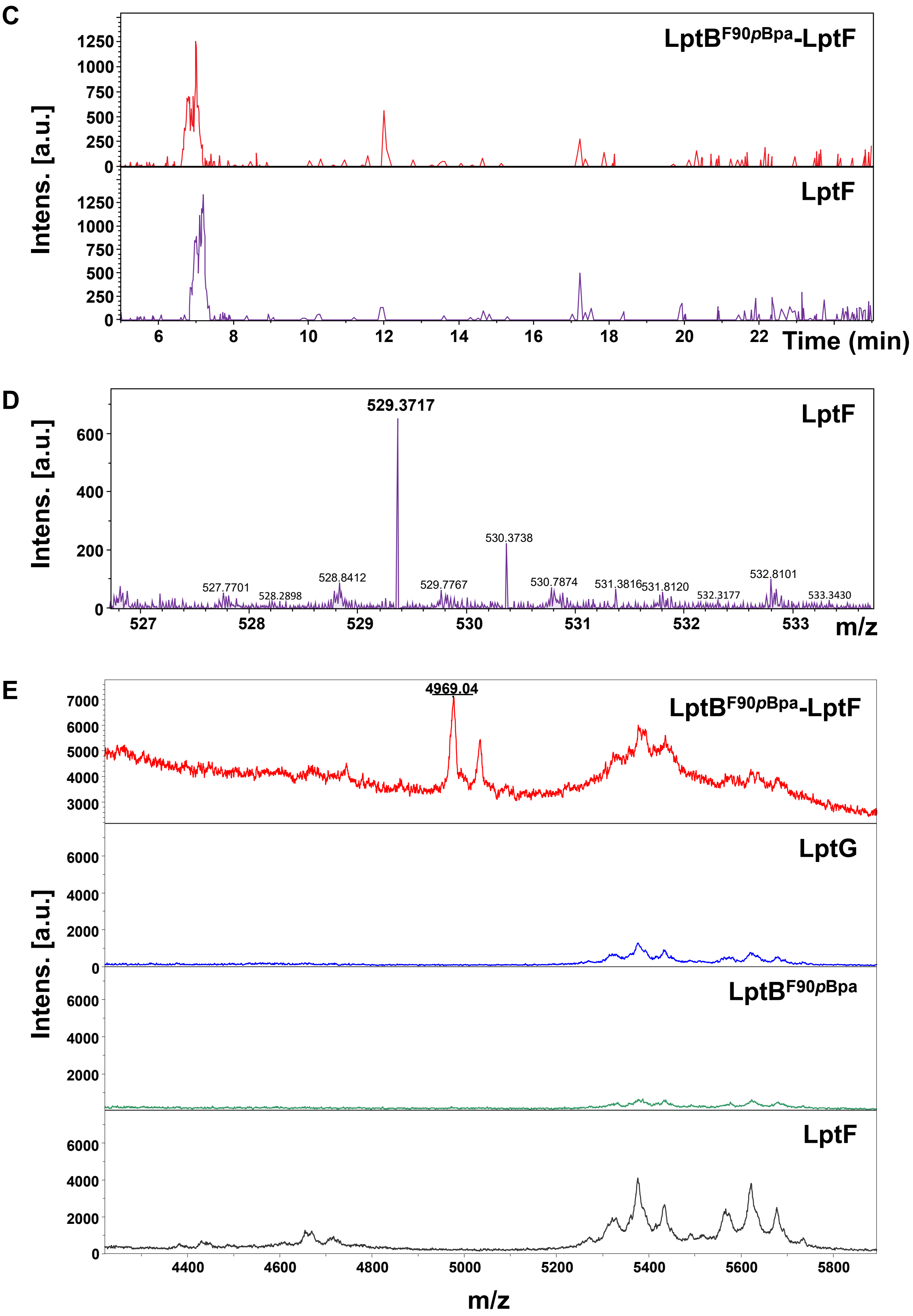
C
Intens. [a.u.]
LptB^{F90pBpa}-LptF
LptF
Time (min)
D
529.3717
LptF
530.3738
527.7701
528.2898
528.8412
529.7767
530.7874
531.3816
531.8120
532.3177
532.8101
533.3430
m/z
E
4969.04
LptB^{F90pBpa}-LptF
LptG
LptB^{F90pBpa}
LptF
m/z
