# Supplementary material for: Identification of Residues in the Lipopolysaccharide ABC Transporter That Coordinate ATPase Activity with Extractor Function
Source: mBio. 2016 Oct 18;7(5):e01729-16. doi: 10.1128/mBio.01729-16 (PMC5082905; doi:10.1128/mBio.01729-16)
Supplement: Figure S7 — Structure-function analysis of LptFG coupling helix variants. (A) Permeability of OM of haploid lptFG mutants measured by disc diffusion assay with four antibiotics as described in Fig. S1A. Strains carry lptFG alleles on pBAD18LptFG3 derivatives. NR2761 was used as the WT lptFG+ control. The sequences of the LptFG coupling helices are shown at the top. (B) Levels of defective variant LptFG proteins in cultures grown overnight. LptF and LptG immunoblot assays of haploid strains for partial loss-of-function LptF (left) and LptG (right) variants, respectively, grown in LB. (C) LptF and LptG immunoblot assays of merodiploid strains for total loss-of-function variants grown in LB and of haploid strains for conditional loss-of-function variants grown in glucose minimal medium. The designation 754 refers to strain NR754, which was used as the control for chromosomally produced LptFG. For haploid strains, the WT is NR2761. For merodiploid strains, the WT is NR3079. Download [file mbo005163035sf7.pdf]

A

LptF 77 G K L **Y** T E S **E** I T V M H **A** **C** G 92
LptG 81 G M **L** **A** Q R S **E** **L** V V M Q A **S** **G** 96

		Zone of inhibition (in mm)			
Relevant alleles	Media	Bacitracin	Novobiocin	Erythromycin	Rifampicin
lptFG⁺	LB	<6	(8)	(13)	10(11)
lptF(Y80A)	LB	8	(8)	(16)	11
lptF(E84A)	LB	15	(8)	(18)	13(20)
lptF(E84D)	LB	17(22)	11(13)	10(24)	11(24)
lptF(A90C)	LB	8(9)	(11)	8(16)	11(12)
lptF(C91A)	LB	9	8(10)	10(14)	11(12)
lptF(C91S)	LB	9(10)	9(11)	10(14)	11(12)
lptG(L83C)	LB	9(11)	10	9(17)	10(14)
lptG(A84Y)	LB	11(16)	13	10(20)	10(15)
lptG(E88A)	LB	10	(14)	11(23)	11(17)
lptG(E88D)	LB	17(19)	17	10(27)	12(27)
lptG(L89C)	LB	11(19)	12	10(22)	11(16)
lptG(S95C)	LB	9(10)	(9)	9(16)	11(12)
lptG(G96A)	LB	11(23)	11	11(18)	10(15)
lptFG⁺	M63gluc	<6	(14)	(13)	10
lptF(E84A) lptG(E88A)	M63gluc	7 (11)	(14)	(20)	17 (24)
lptF(E84D) lptG(E88D)	M63gluc	13	(25)	(23)	16 (26)

B

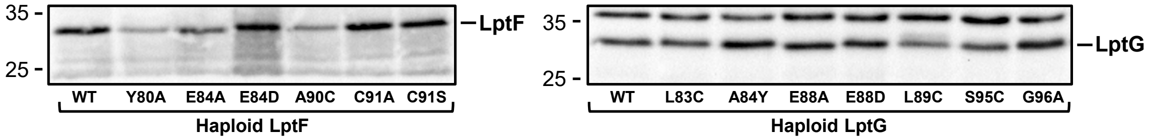

C

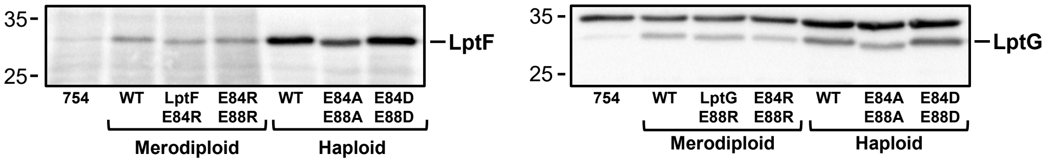

FIG S7
