# Supplementary material for: Identification of Residues in the Lipopolysaccharide ABC Transporter That Coordinate ATPase Activity with Extractor Function
Source: mBio. 2016 Oct 18;7(5):e01729-16. doi: 10.1128/mBio.01729-16 (PMC5082905; doi:10.1128/mBio.01729-16)
Supplement: Table S1 — Strains used in this study. [file mbo005163035st1.docx]

**Table S1. Strains used in this study.**

| Strain | Genotype | Source |
| --- | --- | --- |
| DH5α | F^-^ φ80*lacZ*ΔM15 Δ(*lacZYA-argF*)U169 *recA1 endA1 hsdR17* (r_K_^-^, m_K_^+^) *phoA sup*E44 λ^-^ *thi-*1 | Life Technologies |
| DY378 | W3110 λcI857 Δ(*cro*-*bioA*) | (8) |
| NovaBlue | *endA1* *hsdR17* (r_K_−,m_K_+) *sup*E44 *thi*-1 *recA1* *gyrA96 relA1* *lac* F′[*proA^+^B^+^* *lacI^q^Z*ΔM15::Tn10] | Novagen |
| MC4100 | F^-^ *araD139* Δ(*argF-lac*)U169 *rpsL150 relA1 flbB5301 deoC1 ptsF25 rbsR* | (13) |
| NR754 | MC4100 *ara^+^* | (1) |
| NR2050 | NR754 *tet2* Δ*lptB::frt* (pRC7KanLptB) | (2) |
| NR2101 | NR754 *tet2* Δ*lptB::frt* (pET23/42LptB) | (2) |
| NR2573 | NR754 *tet2* Δ*lptB::frt* (pET23/42LptBF90Y) | (2) |
| NR2575 | NR754 (pET23/42LptBF90A) | (2) |
| NR2583 | NR754 (pET23/42LptB) | This study |
| NR2605 | NR754 *tet2* Δ*lptB::frt* (pET23/42LptBR91A) | This study |
| NR2623 | NR754 *tet2* Δ*lptB::frt* (pET23/42LptBL93E) | This study |
| NR2625 | NR754 *tet2* Δ*lptB::frt* (pET23/42LptBL72E) | This study |
| NR2647 | NR754 *tet2* Δ*lptB::frt* (pET23/42LptBR92E) | This study |
| NR2689 | NR754 *tet2* Δ*lptB::frt* (pET23/42LptBL93R) | This study |
| NR2720 | NR754 *tet2* Δ*lptB::frt* (pET23/42LptBL72R) | This study |
| NR2840 | NR754 *tet2* Δ*lptB::frt* (pET23/42LptBR150A) | This study |
| NR2884 | NR754 *tet2* Δ*lptB::frt* (pET23/42LptBR77A) | This study |
| NR2904 | NR754 *tet2* Δ*lptB::frt* (pET23/42LptBI105A) | This study |
| NR2909 | NR754 *tet2* Δ*lptB::frt* (pET23/42LptBH73A) | This study |
| NR3231 | NR754 *tet2* Δ*lptB::frt* (pET23/42LptBL93F) | This study |
| NR3859 | NR754 *tet2* Δ*lptB::frt* (pET23/42LptBS88A) | This study |
| NR3860 | NR754 *tet2* Δ*lptB::frt* (pET23/42LptBR91E) | This study |
| NR3862 | NR754 *tet2* Δ*lptB::frt* (pET23/42LptBR92A) | This study |
| NR3863 | NR754 *tet2* Δ*lptB::frt* (pET23/42LptBR150K) | This study |
| NR3974 | NR754 *tet2* Δ*lptB::frt* (pET23/42LptBF90I) | This study |
| NR3935 | NR754 (pET23/42LptBR150E) | This study |
| NR2105 | NR754 (pSUP-BpaRS-6TRN) | This study |
| NR2153 | NR754 Δ*lptB::kan* (pET23/42LptBL72Am, pSUP-BpaRS-6TRN) | This study |
| NR2401 | NR754 Δ*lptB::kan* (pET23/42LptBY13Am, pSUP-BpaRS-6TRN) | This study |
| NR2403 | NR754 Δ*lptB::kan* (pET23/42LptBD64Am, pSUP-BpaRS-6TRN) | This study |
| NR2404 | NR754 Δ*lptB::kan* (pET23/42LptBH73Am, pSUP-BpaRS-6TRN) | This study |
| NR2405 | NR754 Δ*lptB::kan* (pET23/42LptBR77Am, pSUP-BpaRS-6TRN) | This study |
| NR2406 | NR754 Δ*lptB::kan* (pET23/42LptBF90Am, pSUP-BpaRS-6TRN) | This study |
| NR2407 | NR754 Δ*lptB::kan* (pET23/42LptBR92Am, pSUP-BpaRS-6TRN) | This study |
| NR2408 | NR754 Δ*lptB::kan* (pET23/42LptBD97Am, pSUP-BpaRS-6TRN) | This study |
| NR2409 | NR754 Δ*lptB::kan* (pET23/42LptBM100Am, pSUP-BpaRS-6TRN) | This study |
| NR2410 | NR754 Δ*lptB::kan* (pET23/42LptBQ104Am, pSUP-BpaRS-6TRN) | This study |
| NR2411 | NR754 Δ*lptB::kan* (pET23/42LptBI105Am, pSUP-BpaRS-6TRN) | This study |
| NR3540 | NR754 Δ*lptB::kan* (pBAD18LptFG3, pCL-His6-LptBF90Am, pSUP-BpaRS-6TRN) | This study |
| NR3720 | NR754 Δ*lptFG::kan* (pBAD18LptFG3, pCL-His6-LptB, pSUP-BpaRS-6TRN) | This study |
| NR3877 | NR754 *tet2* Δ*lptB::frt* (pET23/42LptB, pSUP-BpaRS-6TRN) | This study |
| NR4020 | NR754 Δ*lptB::kan* (pBAD18LptFG3, pCL-His6-LptBR91Am, pSUP-BpaRS-6TRN) | This study |
| KRX | [F´, *traD36*, Δ*ompP*, *proA^+^B^+^*, *lacI^q^*, Δ(*lacZ*)M15] Δ*ompT*, *endA1*, *recA1*, *gyrA96* (Nalr), *thi*-1, *hsdR17* (r_k_^–^, m_k_^+^), e14^–^ (McrA^–^), *relA1*, *supE44*, Δ(*lac*-*proAB*), Δ(*rhaBAD*)::T7 RNA polymerase | Promega |
|  | KRX (pCDFduet-LptBF90Am-LptFG, pET22/42-LptC-His, pSUP-BpaRS-6TRN) | This study |
| NR3401 | NR754 Δ*lptFG::kan* (pBAD18LptFG3/LptFY80Am, pCL-His6-LptB, pSUP-BpaRS-6TRN) | This study |
| NR3402 | NR754 Δ*lptFG::kan* (pBAD18LptFG3/LptFH89Am, pCL-His6-LptB, pSUP-BpaRS-6TRN) | This study |
| NR3707 | NR754 (pCL-His6-LptB) | This study |
| NR3721 | NR754 Δ*lptFG::kan* (pBAD18LptFG3/LptGQ85Am, pCL-His6-LptB, pSUP-BpaRS-6TRN) | This study |
| NR3799 | NR754 Δ*lptFG::kan* (pBAD18LptFG3/LptFT81Am, pCL-His6-LptB, pSUP-BpaRS-6TRN) | This study |
| NR3800 | NR754 Δ*lptFG::kan* (pBAD18LptFG3/LptFS83Am, pCL-His6-LptB, pSUP-BpaRS-6TRN) | This study |
| NR3882 | NR754 Δ*lptFG::kan* (pBAD18LptFG3/LptFC91Am, pCL-His6-LptB, pSUP-BpaRS-6TRN) | This study |
| NR3938 | NR754 Δ*lptFG::kan* (pBAD18LptFG3/LptGS87Am, pCL-His6-LptB, pSUP-BpaRS-6TRN) | This study |
| NR3939 | NR754 Δ*lptFG::kan* (pBAD18LptFG3/LptGS95Am, pCL-His6-LptB, pSUP-BpaRS-6TRN) | This study |
| NR4080 | NR754 Δ*lptFG::kan* (pBAD18LptFG3/LptFV87Am, pCL-His6-LptB, pSUP-BpaRS-6TRN) | This study |
| NR4081 | NR754 Δ*lptFG::kan* (pBAD18LptFG3/LptFM88Am, pCL-His6-LptB, pSUP-BpaRS-6TRN) | This study |
| NR4082 | NR754 Δ*lptFG::kan* (pBAD18LptFG3/LptGV91Am, pCL-His6-LptB, pSUP-BpaRS-6TRN) | This study |
| NR4083 | NR754 Δ*lptFG::kan* (pBAD18LptFG3/LptGQ93Am, pCL-His6-LptB, pSUP-BpaRS-6TRN) | This study |
| NR1958 | NR754 Δ*pyrB::frt* | This study |
| NR2759 | NR754 Δ*lptFG::frt* (pRC7KanLptFG) | This study |
| NR2761 | NR754 Δ*lptFG::frt* (pBAD18LptFG3) | This study |
| NR2762 | NR754 Δ*lptFG::frt* (pBAD18LptFG3/LptGE88A) | This study |
| NR2769 | NR754 Δ*lptFG::frt* (pBAD18LptFG3/LptGS95A) | This study |
| NR2770 | NR754 Δ*lptFG::frt* (pBAD18LptFG3/LptGG96A) | This study |
| NR3079 | NR754 (pBAD18LptFG3) | This study |
| NR3240 | NR754 Δ*lptFG::frt* (pBAD18LptFG3/LptFE82A) | This study |
| NR3241 | NR754 Δ*lptFG::frt* (pBAD18LptFG3/LptFH89A) | This study |
| NR3242 | NR754 Δ*lptFG::frt* (pBAD18LptFG3/LptFC91S) | This study |
| NR3257 | NR754 Δ*lptFG::frt* (pBAD18LptFG3/LptFK78A) | This study |
| NR3258 | NR754 Δ*lptFG::frt* (pBAD18LptFG3/LptFT81A) | This study |
| NR3259 | NR754 Δ*lptFG::frt* (pBAD18LptFG3/LptFS83A) | This study |
| NR3260 | NR754 Δ*lptFG::frt* (pBAD18LptFG3/LptFT86A) | This study |
| NR3261 | NR754 Δ*lptFG::frt* (pBAD18LptFG3/LptFM88A) | This study |
| NR3262 | NR754 Δ*lptFG::frt* (pBAD18LptFG3/LptFC91A) | This study |
| NR3265 | NR754 Δ*lptFG::frt* (pBAD18LptFG3/LptFE84A) | This study |
| NR3307 | NR754 Δ*lptFG::frt* (pBAD18LptFG3/LptGM92A) | This study |
| NR3308 | NR754 Δ*lptFG::frt* (pBAD18LptFG3/LptGS95C) | This study |
| NR3331 | NR754 Δ*lptFG::frt* (pBAD18LptFG3/LptFY80A) | This study |
| NR3327 | NR754 Δ*lptFG::frt* (pBAD18LptFG3/LptFE84A/LptGE88A) | This study |
| NR4129 | NR754 Δ*lptFG::frt* (pBAD18LptFG3/LptGS87C) | This study |
| NR3641 | NR754 Δ*lptFG::frt* (pBAD18LptFG3/LptGL89C) | This study |
| NR3659 | NR754 Δ*lptFG::frt* (pBAD18LptFG3/LptFV87C) | This study |
| NR3660 | NR754 Δ*lptFG::frt* (pBAD18LptFG3/LptGL83C) | This study |
| NR3661 | NR754 Δ*lptFG::frt* (pBAD18LptFG3/LptGA84Y) | This study |
| NR3662 | NR754 Δ*lptFG::frt* (pBAD18LptFG3/LptGV91C) | This study |
| NR3680 | NR754 Δ*lptFG::frt* (pBAD18LptFG3/LptFE84D) | This study |
| NR3681 | NR754 Δ*lptFG::frt* (pBAD18LptFG3/LptGE88D) | This study |
| NR3684 | NR754 Δ*lptFG::frt* (pBAD18LptFG3/LptFL79C) | This study |
| NR3685 | NR754 Δ*lptFG::frt* (pBAD18LptFG3/LptFA90C) | This study |
| NR3687 | NR754 Δ*lptFG::frt* (pBAD18LptFG3/LptGM82C) | This study |
| NR3688 | NR754 Δ*lptFG::frt* (pBAD18LptFG3/LptGQ85C) | This study |
| NR3689 | NR754 Δ*lptFG::frt* (pBAD18LptFG3/LptGR86C) | This study |
| NR3690 | NR754 Δ*lptFG::frt* (pBAD18LptFG3/LptGV90C) | This study |
| NR3691 | NR754 Δ*lptFG::frt* (pBAD18LptFG3/LptGQ93A) | This study |
| NR3692 | NR754 Δ*lptFG::frt* (pBAD18LptFG3/LptGA94C) | This study |
| NR3699 | NR754 (pBAD18LptFG3/LptFE84R) | This study |
| NR3701 | NR754 (pBAD18LptFG3/LptGE88R) | This study |
| NR3737 | NR754 Δ*lptFG::frt* (pBAD18LptFG3/LptFI85C) | This study |
| NR3738 | NR754 Δ*lptFG::frt* (pBAD18LptFG3/LptFG92A) | This study |
| NR3755 | NR754 Δ*lptFG::frt* (pBAD18LptFG3/LptFE84D/LptGE88D) | This study |
| NR3853 | NR754 (pBAD18LptFG3/LptFE84R/LptGE88R) | This study |
| NR760 | NR754 *lptD4213* | (10) |
| NR2719 | NR754 (pET23/42LptBH195A) | (2) |
| NR3587 | NR754 *tet2 lptBR91S* Δ*lptFG::frt* (pRC7KanLptFG) | This study |
| NR3590 | NR754 *tet2 lptBR91S* Δ*lptFG::frt* (pBAD18LptFG3/LptFE84A) | This study |
| NR3592 | NR754 *tet2 lptBR91S* Δ*lptFG::frt* (pBAD18LptFG3/LptGE88A) | This study |
| NR3601 | NR754 *tet2 lptBR91S lptD4213* | This study |
| NR3602 | NR754 *tet2 lptBR91S* | This study |
| NR3642 | NR754 *tet2 lptBR91S* Δ*lptFG::frt* (pBAD18LptFG3/LptGL89C) | This study |
| NR3652 | NR754 (pET23/42LptBR91S/F90A) | This study |
| NR3653 | NR754 (pET23/42LptBR91S/H195A) | This study |
| NR3703 | NR754 *tet2 lptBR91S* Δ*lptFG::frt* (pBAD18LptFG3/LptFE84D) | This study |
| NR3704 | NR754 *tet2 lptBR91S* Δ*lptFG::frt* (pBAD18LptFG3/LptGE88D) | This study |
| NR3705 | NR754 *tet2 lptBR91S* Δ*lptFG::frt* (pBAD18LptFG3/LptFE84R) | This study |
| NR3706 | NR754 *tet2 lptBR91S* Δ*lptFG::frt* (pBAD18LptFG3/LptGE88R) | This study |
| NR3746 | NR754 Δ*lptFG::frt* (pBAD18LptFG3/LptFE84R, pCL-His6-LptBR91E) | This study |
| NR3747 | NR754 Δ*lptFG::frt* (pBAD18LptFG3/LptGE88R, pCL-His6-LptBR91E) | This study |
| NR3749 | NR754 Δ*lptFG::frt* (pBAD18LptFG3/LptFE84R, pCL-His6-LptBR91S) | This study |
| NR3750 | NR754 Δ*lptFG::frt* (pBAD18LptFG3/LptGE88R, pCL-His6-LptBR91S) | This study |
| NR3753 | NR754 Δ*lptFG::frt* (pBAD18LptFG3/LptGE88R, pCL-His6-LptBR91K, pRC7KanLptFG) | This study |
| NR4010 | NR754 Δ*lptFG::frt* (pBAD18LptFG3/LptFE84R, pCL-His6-LptB, pRC7KanLptFG) | This study |
| NR4011 | NR754 Δ*lptFG::frt* (pBAD18LptFG3/LptGE88R, pCL-His6-LptB, pRC7KanLptFG) | This study |
| NR4056 | NR754 Δ*lptFG::frt* (pBAD18LptFG3/LptFE84R, pCL-His6-LptBR91K) | This study |
| NR4127 | NR754 *tet2 lptBR91S* Δ*lptFG::frt* (pBAD18LptFG3/LptFE84A/LptGE88A) | This study |
| NR4130 | NR754 *tet2 lptBR91S* Δ*lptFG::frt* (pBAD18LptFG3/LptFE84D/LptGE88D, pRC7KanLptFG) | This study |
| NR4131 | NR754 *tet2 lptBR91S* Δ*lptFG::frt* (pBAD18LptFG3/LptFE84R/LptGE88R, pRC7KanLptFG) | This study |
|  | NR2761 (pCDFduet-LptB-LptC-His) | This study |
|  | NR2761 (pCDFduet-LptB(R91S)-LptC-His) | This study |
|  | NR3327(pCDFduet-LptB-LptC-His) | This study |
|  | NR3327 (pCDFduet-LptB(R91S)-LptC-His) | This study |
