# Supplementary material for: Identification of Residues in the Lipopolysaccharide ABC Transporter That Coordinate ATPase Activity with Extractor Function
Source: mBio. 2016 Oct 18;7(5):e01729-16. doi: 10.1128/mBio.01729-16 (PMC5082905; doi:10.1128/mBio.01729-16)
Supplement: Table S2 — Primers used in this study. Primers used for the blunt-end site-directed mutagenesis with ligation protocol are marked with asterisks. [file mbo005163035st2.docx]

**Table S2. Primers used in this study. Primers used for blunt-end SDM with ligation protocol are marked with ***

| **Primer name** | **Amino acid change** | **Primer sequence 5’ to 3’** |
| --- | --- | --- |
| 5LptB77up | N/A | GTT CTG GTG CCG TCG CAG |
| 3LptB50down | N/A | CAT GTT CAG AAT CGT ACT CTC CTG C |
| 5LptC154up | N/A | GTT TAA GCG TCG CCG TGG |
| 5LptF79up | N/A | TCA AGA GAA TAA ATG ACG TTT AAG CC |
| 3LptG226down | N/A | GCC AAG TGA CGA ATC AGA TTG |
| BlaP1 | N/A | ATG AGT ATT CAA CAT TTC CGT GTC GCC CTT ATT CCC TTT TTT GCG GCA TTT TGC CTT CCT GTT TTT GCT CGT GTA GGC TGG AGC TGC TTC |
| BlaP2 | N/A | TTA CCA ATG CTT AAT CAG TGA GGC ACC TAT CTC AGC GAT CTG TCT ATT TCG TTC ATC CAT AGT TGC CTG ACA TAT GAA TAT CCT CCT TA |
| EcoR1_5LptFG_SD | N/A | AGC GGA ATT CGA CGA GTT TTT AAG GAG GTA TTT AAA GTG |
| EcoR1_3LptFG_SD | N/A | CGT CGA ATT CCG CTA GCC CAA AAA AAC GG |
| EcoR1_5LptFG_ATG | N/A | AGC GGA ATT CGA CGA GTT TTT AAG GAG GTA TTT AAA ATG ATA ATC ATA AG |
| yhbGN-H-NdeI | N/A | ATA TCA TAT GCA TCA TCA TCA TCA TCA TGG AAT GGC AAC ATT AAC TGC AAA GAA C |
| yhbGC-AvrII | N/A | ACA CCC TAG GTC AGA GTC TGA AGT CTT CCC C |
| LptB-Y13Am-f | Y13Am | TTC TAC CAC GCG ACG GCC TTT CTA GGC TTT TGC AAG GTT |
| LptB-Y13Am-r | Y13Am | AAC CTT GCA AAA GCC TAG AAA GGC CGT CGC GTG GTA GAA |
| LptB-D64Am-f | D64Am | CGG GCA ACA TCA TTA TTG ATT AGG ACG ATA TCA GTC TGC TGC C |
| LptB-D64Am-r | D64Am | GGC AGC AGA CTG ATA TCG TCC TAA TCA ATA ATG ATG TTG CCC G |
| LptB-L72Am-f | L72Am | CGC GCG TGC ATG CTA AGG CAG CAG A |
| LptB-L72Am-r | L72Am | TCT GCT GCC TTA GCA TGC ACG CGC G |
| LptB-L72E-f | L72E | CGC GCG TGC ATG CTC AGG CAG CAG A |
| LptB-L72E-r | L72E | TCT GCT GCC TGA GCA TGC ACG CGC G |
| LptB-L72R-f | L72R | GCG CGT GCA TGC CGA GGC AGC AG |
| LptB-L72R-r | L72R | CTG CTG CCT CGG CAT GCA CGC GC |
| 5LptBH73A | H73A | TCA GTC TGC TGC CTC TGG CGG CAC GCG CGC GCC GC |
| 3LptBH73A | H73A | GCG GCG CGC GCG TGC CGC CAG AGG CAG CAG ACT GA |
| LptB-H73Am-f | H73Am | GCT GCC TCT GTA GGC ACG CGC GC |
| LptB-H73Am-r | H73Am | GCG CGC GTG CCT ACA GAG GCA GC |
| 5LptBR77A | R77A | CAT GCA CGC GCG GCC CGC GGT ATC G |
| 3LptBR77A | R77A | CGA TAC CGC GGG CCG CGC GTG CAT G |
| LptB-R77Am-f | R77Am | ATG CAC GCG CGT AGC GCG GTA TCG G |
| LptB-R77Am-r | R77Am | CCG ATA CCG CGC TAC GCG CGT GCA T |
| 5LptBS88A | S88A | TGC CAC AGG AAG CCG CCA TTT TCC GTC GC |
| 3LptBS88A | S88A | GCG ACG GAA AAT GGC GGC TTC CTG TGG CA |
| 5LptBF90A | F90A | GCC ACA GGA AGC CTC CAT TGC CCG TCG CCT C |
| 3LptBF90A | F90A | GAG GCG ACG GGC AAT GGA GGC TTC CTG TGG C |
| 5LptBF90Am | F90Am | TGC CAC AGG AAG CCT CCA TTT AGC GTC GCC TCA G |
| 3LprBF90Am | F90Am | CTG AGG CGA CGC TAA ATG GAG GCT TCC TGT GGC A |
| LptBF90I | F90I | GCC ACA GGA AGC CTC CAT TAT CCG TCG CCT |
| LptBF90I_antisense | F90I | AGG CGA CGG ATA ATG GAG GCT TCC TGT GGC |
| LptBF90Y | F90Y | CCA CAG GAA GCC TCC ATT TAC CGT CGC CTC |
| LptBF90Y_antisense | F90Y | GAG GCG ACG GTA AAT GGA GGC TTC CTG TGG |
| 5LptBR91A | R91A | ACA GGA AGC CTC CAT TTT CGC TCG CCT CAG CGT T |
| 3LptBR91A | R91A | AAC GCT GAG GCG AGC GAA AAT GGA GGC TTC CTG T |
| LptBR91Amsense | R91Am | CAG GAA GCC TCC ATT TTC TAG CGC CTC AGC G |
| LptBR91Amanti | R91Am | CGC TGA GGC GCT AGA AAA TGG AGG CTT CCT G |
| LptBR91Esense | R91E | GAA GCC TCC ATT TTC GAG CGC CTC AGC GTT TAC |
| LptBR91Eanti | R91E | GTA AAC GCT GAG GCG CTC GAA AAT GGA GGC TTC |
| LptBR91Ksense | R91K | GAA GCC TCC ATT TTC AAG CGC CTC AGC GTT TAC |
| LptBR91Kanti | R91K | GTA AAC GCT GAG GCG CTT GAA AAT GGA GGC TTC |
| LptBR91Ssense | R91S | ACA GGA AGC CTC CAT TTT CAG TCG CCT CAG CGT T |
| LptBR91Santi | R91S | AAC GCT GAG GCG ACT GAA AAT GGA GGC TTC CTG T |
| 5LptBR92A | R92A | AGG AAG CCT CCA TTT TCC GTG CCC TCA GCG TTT AC |
| 3LptBR92A | R92A | GTA AAC GCT GAG GGC ACG GAA AAT GGA GGC TTC CT |
| LptB-R92Am-f | R92Am | CTC CAT TTT CCG TTA GCT CAG CGT TTA CG |
| LptB-R92Am-r | R92Am | CGT AAA CGC TGA GCT AAC GGA AAA TGG AG |
| 5LptBR92E | R92E | CAG GAA GCC TCC ATT TTC CGT GAA CTC AGC GTT TAC GAT AAC CTG |
| 3LptBR92E | R92E | CAG GTT ATC GTA AAC GCT GAG TTC ACG GAA AAT GGA GGC TTC CTG |
| LptB-L93E-f | L93E | GGT TAT CGT AAA CGC TCT CGC GAC GGA AAA TGG A |
| LptB-L93E-r | L93E | TCC ATT TTC CGT CGC GAG AGC GTT TAC GAT AAC C |
| 5LptBL93F | L93F | CCT CCA TTT TCC GTC GCT TCA GCG TTT ACG ATA AC |
| 3LptBL93F | L93F | GTT ATC GTA AAC GCT GAA GCG ACG GAA AAT GGA GG |
| LptB-L93R-f | L93R | GGT TAT CGT AAA CGC TGC GGC GAC GGA AAA TGG A |
| LptB-L93R-r | L93R | TCC ATT TTC CGT CGC CGC AGC GTT TAC GAT AAC C |
| LptB-D7Am-f | D97Am | CGC CTC AGC GTT TAC TAG AAC CTG ATG GCG GTA C |
| LptB-D97Am-r | D97Am | GTA CCG CCA TCA GGT TCT AGT AAA CGC TGA GGC G |
| LptB-M100Am-f | M100Am | GTT TAC GAT AAC CTG TAG GCG GTA CTG CAA ATT |
| LptB-M100Am-r | M100Am | AAT TTG CAG TAC CGC CTA CAG GTT ATC GTA AAC |
| LptB-Q104Am-f | Q104Am | ATG GCG GTA CTG TAG ATT CGT GAC GAC |
| LptB-Q104Am-r | Q104Am | GTC GTC ACG AAT CTA CAG TAC CGC CAT |
| 5LptBI105A | I105A | GCG GTA CTG CAA GCG CGT GAC GAC TTG TC |
| 3LptBI105A | I105A | GAC AAG TCG TCA CGC GCT TGC AGT ACC GC |
| LptB-I105Am-f | I105Am | GAT GGC GGT ACT GCA ATA GCG TGA CGA CTT GTC T |
| LptB-I105Am-r | I105Am | AGA CAA GTC GTC ACG CTA TTG CAG TAC CGC CAT C |
| 5LptBR150A | R150A | GTG TAG AAA TTG CCG CGG CAC TGG CTG CGA |
| 3LptBR150A | R150A | TCG CAG CCA GTG CCG CGG CAA TTT CTA CAC |
| LptBR150Esense | R150E | GTG TAG AAA TTG CCG AGG CAC TGG CTG CG |
| LptBR150Eanti | R150E | CGC AGC CAG TGC CTC GGC AAT TTC TAC AC |
| LptBR150Ksense | R150K | CGT GTA GAA ATT GCC AAG GCA CTG GCT GCG |
| LptBR150Kanti | R150K | CGC AGC CAG TGC CTT GGC AAT TTC TAC ACG |
| 5LptFK78A | K78A | CTG CTG ATG ACG CTG GGC GCA CTG TAT ACC GAA AGT GA |
| 3LptFK78A | K78A | TCA CTT TCG GTA TAC AGT GCG CCC AGC GTC ATC AGC AG |
| LptFL79Csense | L79C | GAC GCT GGG CAA ATG TTA TAC CGA AAG TG |
| LptFL79Canti | L79C | CAC TTT CGG TAT AAC ATT TGC CCA GCG TC |
| 5LptFY80A | Y80A | GAT GAC GCT GGG CAA ACT GGC TAC CGA AAG TGA AAT TAC G |
| 3LptFY80A | Y80A | CGT AAT TTC ACT TTC GGT AGC CAG TTT GCC CAG CGT CAT C |
| LptFY80Amsense | Y80Am | GCT GGG CAA ACT GTA GAC CGA AAG TGA |
| LptFY80Amanti | Y80Am | TCA CTT TCG GTC TAC AGT TTG CCC AGC |
| 5LptFT81A | T81A | GAT GAC GCT GGG CAA ACT GTA TGC CGA AAG TGA AAT |
| 3LptFT81A | T81A | ATT TCA CTT TCG GCA TAC AGT TTG CCC AGC GTC ATC |
| LptFT81Amsense | T81Am | GAC GCT GGG CAA ACT GTA TTA GGA AAG TGA AAT TAC GGT AAT G |
| LptFT81Amanti | T81Am | CAT TAC CGT AAT TTC ACT TTC CTA ATA CAG TTT GCC CAG CGT C |
| 5LptFE82A | E82A | CTG GGC AAA CTG TAT ACC GCA AGT GAA ATT ACG GTA ATG |
| 3LptFE82A | E82A | CAT TAC CGT AAT TTC ACT TGC GGT ATA CAG TTT GCC CAG |
| 5LptFS83A | S83A | CGC TGG GCA AAC TGT ATA CCG AAG CTG AAA TTA CGG TAA TG |
| 3LptFS83A | S83A | CAT TAC CGT AAT TTC AGC TTC GGT ATA CAG TTT GCC CAG CG |
| LptFS83Amsense | S83Am | CTG GGC AAA CTG TAT ACC GAA TAG GAA ATT ACG GTA ATG C |
| LptFS83Amanti | S83Am | GCA TTA CCG TAA TTT CCT ATT CGG TAT ACA GTT TGC CCA G |
| 5LptFE84A | E84A | CAA ACT GTA TAC CGA AAG TGC AAT TAC GGT AAT GCA TGC CT |
| 3LptFE84A | E84A | AGG CAT GCA TTA CCG TAA TTG CAC TTT CGG TAT ACA GTT TG |
| LptFE84Dsense | E84D | GCA AAC TGT ATA CCG AAA GTG ATA TTA CGG TAA TGC |
| LptFE84Danti | E84D | GCA TTA CCG TAA TAT CAC TTT CGG TAT ACA GTT TGC |
| LptFE84Rsense | E84R | GCA AAC TGT ATA CCG AAA GTC GTA TTA CGG TAA TGC |
| LptFE84Ranti | E84R | GCA TTA CCG TAA TAC GAC TTT CGG TAT ACA GTT TGC |
| LptFI85Cfor | I85C | TGT ACG GTA ATG CAT GCC TGC GGC CTG AGC |
| LptFI85Crev | I85C | TTC ACT TTC GGT ATA CAG TTT GCC CAG CG |
| 5LptFT86A | T86A | GGC AAA CTG TAT ACC GAA AGT GAA ATT GCG GTA ATG CAT GCC |
| 3LptFT86A | T86A | GGC ATG CAT TAC CGC AAT TTC ACT TTC GGT ATA CAG TTT GCC |
| LptFV87Amsense | V87Am | GAA AGT GAA ATT ACG TAG ATG CAT GCC TGC GGC |
| LptFV87Amanti | V87Am | GCC GCA GGC ATG CAT CTA CGT AAT TTC ACT TTC |
| LptFV87Cfor* | V87C | CAT GCC TGC GGC CTG AGC AAA GCG |
| LptFV87Crev* | V87C | CAT GCA CGT AAT TTC ACT TTC GGT ATA CAG TTT GCC C |
| 5LptFM88A | M88A | TAC CGA AAG TGA AAT TAC GGT AGC GCA TGC CTG CGG CC |
| 3LptFM88A | M88A | GGC CGC AGG CAT GCG CTA CCG TAA TTT CAC TTT CGG TA |
| LptFM88Amsense | M88Am | CCG AAA GTG AAA TTA CGG TAT AGC ATG CCT GCG GCC TGA GC |
| LptFM88Amanti | M88Am | GCT CAG GCC GCA GGC ATG CTA TAC CGT AAT TTC ACT TTC GG |
| 5LptFH89A | H89A | CGA AAG TGA AAT TAC GGT AAT GGC TGC CTG CGG CCT GA |
| 3LptFH89A | H89A | TCA GGC CGC AGG CAG CCA TTA CCG TAA TTT CAC TTT CG |
| LptFH89Amsense | H89Am | GTG AAA TTA CGG TAA TGT AGG CCT GCG G |
| LptFH89Amanti | H89Am | CCG CAG GCC TAC ATT ACC GTA ATT TCA C |
| LptFA90Cfor* | A90C | CAT TGT TGC GGC CTG AGC AAA GCG GTT CTG |
| LptFA90Crev* | A90C | CAT TAC CGT AAT TTC ACT TTC GGT ATA CAG TTT GC |
| 5LptFC91A | C91A | ACG GTA ATG CAT GCC GCC GGC CTG AGC AAA GC |
| 3LptFC91A | C91A | GCT TTG CTC AGG CCG GCG GCA TGC ATT ACC GT |
| LptFC91Amsense | C91Am | CGG TAA TGC ATG CCT AGG GCC TGA GCA AAG CGG TTC TG |
| LptFC91Amanti | C91Am | CAG AAC CGC TTT GCT CAG GCC CTA GGC ATG CAT TAC CG |
| 5LptFC91S | C91S | ATT ACG GTA ATG CAT GCC TCC GGC CTG AGC |
| 3LptFC91S | C91S | GCT CAG GCC GGA GGC ATG CAT TAC CGT AAT |
| LptFG92Afor* | G92A | TGC GCT CTG AGC AAA GCG GTT CTG GTG |
| LptFG92Arev* | G92A | GGC ATG CAT TAC CGT AAT TTC ACT TTC GGT ATA CAG |
| LptGM82Csense | M82C | TTG CTT GGT CTT GGG TGT CTG GCG CAG |
| LptGM82Canti | M82C | CTG CGC CAG ACA CCC AAG ACC AAG CAA |
| LptGL83Cfor* | L83C | GCA CAG CGC AGC GAA CTG GTG GTG |
| LptGL83Crev* | L83C | ACA CAT CCC AAG ACC AAG CAA CGC |
| LptGA84Ysense | A84Y | CTT GGG ATC TGT ATC AGC GCA GCG AAC |
| LptGA84Yanti | A84Y | GTT CGC TGC GCT GAT ACA GCA TCC CAA G |
| LptGQ85Amsense | Q85Am | GAT GCT GGC GTA GCG CAG CGA ACT G |
| LptGQ85Amanti | Q85Am | CAG TTC GCT GCG CTA CGC CAG CAT C |
| LptGQ85Csense | Q85C | GAT GCT GGC GTG TCG CAG CGA ACT G |
| LptGQ85Canti | Q85C | CAG TTC GCT GCG ACA CGC CAG CAT C |
| LptGR86Csense | R86C | GAT GCT GGC GCA GTG CAG CGA ACT GGT GGT G |
| LptGR86Canti | R86C | CAC CAC CAG TTC GCT GCA CTG CGC CAG CAT C |
| LptGS87Amsense | S87Am | GGG ATG CTG GCG CAG CGC TAG GAA CTG GTG GTG ATG CAG |
| LptGS87Amanti | S87Am | CTG CAT CAC CAC CAG TTC CTA GCG CTG CGC CAG CAT CCC |
| LptGS87Cfor* | S87C | CTG CGA ACT GGT GGT GAT GCA GGC TTC |
| LptGS87Crev* | S87C | CGC TGC GCC AGC ATC CCA AGA CC |
| 5LptGE88A | E88A | CGC AGC GCG CTG GTG GTG ATG CAG GCT TC |
| 3LptGE88A | E88A | CTG CGC CAG CAT CCC AAG AC |
| LptGE88Dsense | E88D | GCA GCG CAG CGA TCT GGT GGT GAT G |
| LptGE88Danti | E88D | CAT CAC CAC CAG ATC GCT GCG CTG C |
| LptGE88Rsense | E88R | GCA GCG CAG CCG TCT GGT GGT GAT G |
| LptGE88Ranti | E88R | CAT CAC CAC CAG ACG GCT GCG CTG C |
| LptGL89Csense | L89C | CGC AGC GAA TGT GTG GTG ATG CAG GC |
| LptGL89Canti | L89C | GCC TGC ATC ACC ACA CAT TCG CTG CG |
| LptGV90Csense | V90C | CGC AGC GAA CTG TGT GTG ATG CAG G |
| LptGV90Canti | V90C | CCT GCA TCA CAC ACA GTT CGC TGC G |
| LptGV91Amsense | V91Am | CAG CGA ACT GGT GTA GAT GCA GGC TTC TGG |
| LptGV91Amanti | V91Am | CCA GAA GCC TGC ATC TAC ACC AGT TCG CTG |
| LptGV91Csense | V91C | GCG AAC TGG TGT GTA TGC AGG CTT CTG |
| LptGV91Canti | V91C | CAG AAG CCT GCA TAC ACA CCA GTT CGC |
| LptGM92Asense | M92A | GAA CTG GTG GTG GCA CAG GCT TCT GG |
| LptGM92Aanti | M92A | CCA GAA GCC TGT GCC ACC ACC AGT TC |
| LptGQ93Amsense | Q93Am | GGT GGT GAT GTA GGC TTC TGG TTT TAC C |
| LptGQ93Amanti | Q93Am | GGT AAA ACC AGA AGC CTA CAT CAC CAC C |
| LptGQ93Csense | Q93C | GGT GGT GAT GTG TGC TTC TGG TTT TAC C |
| LptGQ93Canti | Q93C | GGT AAA ACC AGA AGC ACA CAT CAC CAC C |
| LptGA94Csense | A94C | GTG GTG ATG CAG TGT TCT GGT TTT ACC |
| LptGA94Canti | A94C | GGT AAA ACC AGA ACA CTG CAT CAC CAC |
| LptG_S95A | S95A | GGT GGT GAT GCA GGC TGC TGG TTT TAC CCG TAT |
| LptG_S95A_antisense | S95A | ATA CGG GTA AAA CCA GCA GCC TGC ATC ACC ACC |
| LptGS95Amsense | S95Am | CTG GTG GTG ATG CAG GCT TAG GGT TTT ACC CGT ATG CAG |
| LptGS95Amanti | S95Am | CTG CAT ACG GGT AAA ACC CTA AGC CTG CAT CAC CAC CAG |
| LptGS95Csense | S95C | GGT GAT GCA GGC TTG CGG TTT TAC CCG |
| LptGS95Canti | S95C | CGG GTA AAA CCG CAA GCC TGC ATC ACC |
| LptG_G96A | G96A | GTG ATG CAG GCT TCT GCT TTT ACC CGT ATG CAG |
| LptG_G96A_antisense | G96A | CTG CAT ACG GGT AAA AGC AGA AGC CTG CAT CAC |
| His-Thromb-LptC-Gibrev | N/A | GGT GGT GGT GGC TGC CGC GCG GTA CCA GAG GCT GAG TTT GTT TGT TTT GAA TTT CAT AGG |
| LptC-Thromb-His-Gibf | N/A | CTG GTA CCG CGC GGC AGC CAC CAC CAC CAC CAC CAC CAC CAC |
| pET22/42-upstrm-Gibrev | N/A | CCC CTA TAG TGA GTC GTA TTA ATT TCG CGG GAT CGA GAT C |
| pET22/42-upstrm-Gibf | N/A | CGC GAA ATT AAT ACG ACT CAC TAT AGG GG |
| LptC-his-fix-fwd | N/A | GTA CCG CGC GGC AGC CAC CAC CAC CAC CAC CAC CAC TAA CTC GAG TCT GGT AAA GAA ACC |
| LptC-his-fix-rev | N/A | GAG TTA GTG GTG GTG GTG GTG GTG GTG GCT GCC GCG CGG TAC CAG AGG CTG AGT TTG |
| LptB-F90Am-f | F90Am | CAG GAA GCC TCC ATT TAG CGT CGC CTC AGC GTT |
| LptB-F90Am-r | F90Am | AAC GCT GAG GCG ACG CTA AAT GGA GGC TTC CTG |
| LptB_R91S_f | R91S | CTG CCA CAG GAA GCC TCC ATT TTC AGC CGC CTC AGC GTT TAC GAT AAC CTG |
| LptB_R91S_r | R91S | CAG GTT ATC GTA AAC GCT GAG GCG GCT GAA AAT GGA GGC TTC CTG TGG CAG |
