# Supplementary material for: Identification of Residues in the Lipopolysaccharide ABC Transporter That Coordinate ATPase Activity with Extractor Function
Source: mBio. 2016 Oct 18;7(5):e01729-16. doi: 10.1128/mBio.01729-16 (PMC5082905; doi:10.1128/mBio.01729-16)
Supplement: Text S1 — Supplemental materials and methods used in this study. Download [file mbo005163035s1.docx]

**SUPPLEMENTAL MATERIAL**

**MATERIALS AND METHODS**

**Strains and growth conditions.** All strains are listed in Table S1. Construction of null alleles for Δ*lptB::kan* and Δ*lptB::frt* and for Δ*lptFG::kan* and Δ*lptFG::frt* were previously described (1, 2). P1_vir_ transduction was used to transfer chromosomal alleles (3). Cells were grown at 37°C with aeration in either LB or M63 minimal media supplemented with 0.2% (wt/vol) glucose. Plates and top agar were prepared with 1.5% and 0.75% w/v agar, respectively. When applicable, ampicillin (125 μg/mL), chloramphenicol (20 μg/mL), kanamycin (30 μg/mL), spectinomycin (50 μg/mL), tetracycline (25μg/mL), carbenicillin (50 μg/mL), X-Gal (33 μg/mL), isopropyl-β-D-1-thiogalactopyranoside (IPTG, 0.16 mM), L-rhamnose (0.02%, wt/vol), and *p*-benzoyl phenylalanine (*p*BPA, 0.48 mM in 1N NaOH) were added to the media.

**Mutant allele construction.** All primers are listed in Table S4. Plasmids were built using restriction enzymes and T4 DNA ligase from New England Biolabs. Construction of pET23/42LptB and pRC7KanLptB were previously described (2). To build pET23/42His6-LptB, chromosomal *lptB* was amplified with primers yhbGN-H-NdeI and yhbGC-AvrII, which added the codons for MHHHHHHG to the 5’ end of *lptB*. The resulting PCR product and pET23/42 plasmid (4) were cut with NdeI and AvrII and ligated together. To construct pCL-His6-LptB, the *his6-lptB* was excised from pET23/42His6-LptB with NdeI and AvrII and ligated with pCL-1 (5) that had been digested with NdeI and AvrII.

Plasmid pBAD18LptFG3 was derived from pBADyjgPQ1 (1) by changing the ribosomal-binding site (RBS) and start codon controlling expression of *lptFG*. First, the native RBS of *lptFG* present in pBADyjgPQ1 was changed for the more optimal AGGAGG RBS (located 14-8 bp upstream of the native GTG start codon) using SDM PCR (conditions described below) with primers EcoR1_5LptFG_SD and EcoR1_3LptFG_SD. The resulting PCR product was digested with EcoRI and self-ligated using T4 DNA ligase (New England Biolabs). The resulting pBADLptFG2 plasmid was used as a template in a subsequent SDM PCR using primers EcoR1_5LptFG_ATG and EcoR1_3LptFG_SD. The resulting PCR product was digested with EcoRI and self-ligated using T4 DNA ligase to generate pBAD18LptFG3.

To construct pRC7LptFG, pBADyjgPQ1 (1) was digested with EcoRI and HindIII. The resulting 2.3 Kbp fragment encoding *lptFG* was inserted into EcoRI-Hind III-digested pRC7 (6) to construct pRC7LptFG. The *bla* gene encoding for ampicillin resistance in pRC7 was replaced with the kanamycin resistance cassette (*kan*) from pKD4 (7) using recombineering as follows. Primers BlaP1 and BlaP2 were used to amplify using PCR the *kan* cassette from pKD4 (7). The resulting PCR product was electroporated into recombineering strain DY378 (8) carrying pRC7LptFG and kanamycin-resistant recombinants were selected at 30 ^o^C on LB agar containing kanamycin. Replacement of *bla* for *kan* was confirmed and the resulting recombinant plasmid was named pRC7KanLptFG.

Mutant alleles of *lptB* were constructed in pET23/42LptB except for those carrying amber substitutions (described below). Mutant alleles of *lptF* and *lptG* were generated in pBAD18LptFG3. Site-directed mutagenesis (SDM) with PfuTurbo (Agilent Technologies, Inc.) or KOD Hot Start DNA Polymerase was used to generate plasmids carrying mutant alleles. The majority of primers were designed according to the QuikChange protocol (Agilent). When indicated, primers that generate blunt-end, linear products were used. These PCR products were phosphorylated with T4 polynucleotide kinase (New England Biolabs) and self-ligated with T4 DNA ligase. All SDM PCRs were digested with DpnI and electroporated into DH5α or NovaBlue competent cells. Plasmids were confirmed by DNA sequence.

Plasmids for purification of LptBFGC-His and *in vitro* assays were modified from the previously described pCDFduet-LptB-LptFG and pET22/42-LptC (2). pET22/42-LptC-His was constructed in two steps. First, *lptC* was amplified from pET22/42-LptC using primers pET22/42-upstrm-Gibf and His-Thromb-LptC-Gibrev, and the vector backbone was amplified using LptC-Thromb-His-Gibf and pET22/42-upstrm-Gibrev. The two products were digested with DpnI, purified (QIAquick PCR Purification Kit, Qiagen), and then ligated together with Gibson Assembly Master Mix (New England Biolabs). To construct pCDFduet-LptB-LptC-His, both pCDFduet-LptB-LptFG and pET22/42-LptC-His were digested with NdeI and KpnI to excise the *lptFG* and *lptC-his* genes respectively, and the LptC-His fragment was ligated into the pCDFduet-LptB fragment using T4 DNA ligase. The location of the KpnI site in the region coding the His tag of LptC prevented the transfer of the tag. Site-directed mutagenesis with primers LptC-his-fix-fwd and LptC-his-fix-rev was used to reinsert the tag. Finally, mutant alleles of *lptB* were generated using site-directed mutagenesis as described above.

**Construction of LptB and LptFG characterization strains.** To construct an LptFG characterization strain, pRC7KanLptFG was brought into the auxotrophic strain NR754 Δ*pyrB::frt*, which cannot grow on glucose minimal medium. The plasmid pRC7KanLptFG was maintained and its presence monitored with the addition of kanamycin, IPTG, and X-gal. The Δ*lptFG::frt* allele was brought in by co-transduction with *pyrB^+^* into the NR754 Δ*pyrB::frt* (pRC7KanLptFG) strain to restore the ability to grow on glucose minimal medium. Presence of the Δ*lptFG::frt* allele was confirmed by the inability to lose pRC7KanLptFG on media lacking kanamycin and by PCR. The resulting strain was named NR2759.

**Construction of cross-linking strains.** To build pET23/42LptB and pET23/42His6-LptB cross-linking strains, first pSUP-BpaRS-6TRN was introduced into NR754 to generate NR2105. Amber-codon variants encoded on pET23/42His6-LptB were introduced into NR2105. Finally the Δ*lptB::kan* allele was brought in by transduction by selecting on glucose M63 plates containing kanamycin and *p*BPA. We were unable to get Δ*lptB::kan* transductants for the crosslinking strain containing His6-LptBL^72^*^p^*^BPA^. However, the allele encoding untagged LptBL^72^*^p^*^BPA^ on the pET23/42LptB plasmid complemented the chromosomal *lptB* deletion. Therefore, for this variant the haploid untagged version was used.

To detect the interacting partner of LptB^F90^*^p^*^BPA^, LptFG levels needed to be increased to a level that is readily detectable by immunoblot. For this purpose, the compatible vectors pBAD18LptFG and pCL-His6-LptB were used to perform LptFG crosslinking. While the origins of pBAD18LptFG3, pCL-His6-LptB, and pSUP-BpaRS-6TRN were previously reported to be compatible with each other, we did experience occasional loss of at least one of the plasmids and their associated resistance markers in strains with this three plasmid combination (9). Therefore, selection for all three plasmids was maintained at all times with the addition of ampicillin, chloramphenicol and spectinomycin.

To build pCL-His6-LptB *p*BPA variants, first the amber codon containing pCL-His6-LptB variants were introduced into NR2105. Next pBAD18LptFG3 was introduced into the resulting strains and chromosomal *lptB* was deleted by transducing Δ*lptB::kan*. To build LptFG cross-linking strains, first pCL-His6-LptB was introduced into NR754 to generate NR3707. LptFG amber variants encoded on pBAD18LptFG3 were then introduced into NR3707. Next, pSUP-BpaRS-6TRN was introduced and chromosomal *lptFG* was deleted by transducing Δ*lptFG::kan*.

**Suppressor analysis of *lptF(E84)* and *lptG(E88)* mutants.** Several cultures of haploid *lptF(E84A) lptG(E88A)* strain NR3327 were grown overnight in LB. Owing to Lpt defects, these cultures only reached an OD_600_ of 0.4-0.9. From each culture, 100-400μL were plated on to LB agar containing bacitracin (50 or 100 μg/mL).

The previously reported marker *tet2*, a mini-Tn*tet* transposon inserted into the chromosome 23 Kb downstream of the *lptCAB* operon, was used to check for linkage to *lptCAB* by P1vir transduction (2). The suppressor phenotype of eight independent suppressors was consistently ~50% linked to *tet2*, suggesting the suppressor mutations were located in the *lptCAB* operon. PCR was performed to amplify the chromosomal locus either extending from 154 bp upstream of *lptC* to 50 bp downstream of *lptB* or 77bp upstream to 50bp downstream of *lptB*. The resulting PCR products were sequenced. In the eight *tet2*-linked suppressors, we found a single base-pair missense mutation in *lptB*; two suppressors had a C271A mutation that resulted in an R91S substitution, five suppressors had a G272A mutation that resulted in an *lptB(R91H)* allele, and one suppressor had a C271T mutation resulting in *lptB(R91C)*.

To confirm that these *lptB* mutations conferred the suppressor phenotype, co-transduction was used to bring *tet2* and the mutant *lptB(R91S)* alleles into NR2759 and the presence of the mutant alleles was confirmed by sequencing. The plasmid pBAD18LptFG3/LptFE84A/LptGE88A was introduced into the resulting strain, NR3587, and *lptFG* haploid strains (i.e. had lost pRC7KanLptFG) were isolated and characterized. We confirmed that the resulting strains behaved phenotypically identical to the original suppressor strains with respect to suppression and OM permeability.

To combine other *lptF(E84)* and *lptG(E88)* alleles with *lptBR91S*, pBAD18LptFG3-derived plasmids with the appropriate *lptF* and *lptG* mutations were introduced into NR3587. Characterization of complementation and OM permeability were compared to NR2759-derived strains carrying wild-type chromosomal *lptB*. To combine *lptB(R91S)* with defective LptB substitutions, mutations were introduced by SDM into pET23/42LptB and the resulting plasmids were characterized in NR2050 as described above. To combine *lptB(R91S)* with *lptD4213*, the *lptB(R91S)* allele was co-transduced with *tet2* into NR760 which contains the *lptD4213* chromosomal allele (10, 11). Presence of the *lptB(R91S)* allele was confirmed by DNA sequencing.

**Over-expression and purification of LptBFGC-His for *in vitro* photo-cross-linking.** The method used for purifying LptB^F90^*^p^*^BPA^FG-LptC-His_7_ complexes is modified from Sherman *et al* (2). Overnight cultures of *E. coli* KRX strain containing plasmids pSup-BpaRS-6TRN, pCDFduet-LptB(F90Am)-LptFG, pET22/42-LptC-His were diluted into 3 L of fresh LB containing 0.63 mM *p*BPA and appropriate antibiotics, grown at 37°C to OD_600_ ~ 0.8, then cooled to 18°C and grown to OD_600_ ~1.2. Expression was induced by addition of 0.02% w/v L-rhamnose monohydrate (Sigma) and 100 μM IPTG, and allowed to proceed 15 h.

Cells were harvested by centrifugation at 4,200 x g for 20 min, resuspended in lysis buffer (50 mM Tris-HCl pH 7.4, 300 mM NaCl, 1 mM MgCl_2_) supplemented with 1 mM PMSF, 100 μg/mL lysozyme and 100 μg/mL DNaseI and homogenized with an IKA T18 basic UltraTurrax. Cells were then lysed by three passes through an EmulsiFlex-C3 cell disrupter at 15,000 psi. Unbroken cells and other debris were removed at 10,000 x g for 10 min, and then membranes were isolated by centrifugation at 100,000 x g for 1 h. Membranes were resuspended in buffer (20 mM Tris-HCl pH 7.4, 300 mM NaCl, 5 mM MgCl_2_, and 15 % glycerol), homogenized by UltraTurrax, supplemented with 2 mM ATP and 1% (wt/vol) n-dodecyl-β-D-maltopyranoside (DDM, Anatrace), and then solubilized at 4°C for 2 h. Insoluble debris were removed by another centrifugation step, 30min at 100,000 x g. The supernatant was collected, supplemented with 2 mM imidazole, and rocked for 1 h with TALON Superflow metal affinity resin (Clontech) pre-equilibrated with affinity buffer [20 mM Tris-HCl pH 7.4, 300 mM NaCl, 0.05% (wt/vol) DDM, and 15% (vol/vol) glycerol]. After allowing it to drain, the resin was washed with 20 column volumes (cv) affinity buffer plus 2 mM imidazole, 10 cv affinity buffer plus 10 mM imidazole, and protein eluted with 8 cv affinity buffer + 100 mM imidazole. The eluate was then concentrated with a 100 kDa molecular weight cutoff Amicon Ultra centrifugal filter (Millipore) and further purified via size-exclusion chromatography using a Superdex 200 10/300GL column (GE Healthcare) in 20 mM Tris-HCl pH 7.4, 300 mM NaCl, 0.05% (wt/vol) DDM, and 10 % (vol/vol) glycerol. Peak fractions were pooled and used immediately or flash-frozen in liquid nitrogen and stored at -80°C for later use.

***In vitro* photo-cross-linking and trypsin digestion for mass spectrometry analysis.** To produce cross-linked complexes LptB^F90^*^p^*^BPA^FG-LptC-His protein solution was UV-irradiated at 365 nm on ice for 1 h, and then either frozen -80°C or immediately mixed with 2x SDS loading dye [100 mM Tris-HCl pH 6.8, 4% (w/v) SDS, 0.05% (w/v) bromothymol blue, 20%v/v glycerol, and 5% (vol/vol) β-mercaptoethanol], boiled for 10 min and loaded onto a 14% polyacrylamide gel and visualized with Coomassie blue stain (adapted from (5)). To verify the identity of bands produced by UV irradiation, transfer to PVDF membrane and immunoblotting against all IM Lpt proteins was carried out as described above.

Coomassie-stained bands corresponding to LptB^F90^*^p^*^BPA^, LptF, LptG and LptB^F90^*^p^*^BPA^-LptF were excised from the polyacrylamide gel and chopped into small pieces (~1 mm^3^). Gel pieces were destained by covering them with 100 μL 25 mM NH_4_HCO_3_/50% acetonitrile, vortexing 10 min at room temperature, removing supernatant and repeating until the slices were destained. If gel slices would not fully destain, they were heated to 70°C in 25 mM NH_4_HCO_3_/50% acetonitrile. After discarding the supernatant and drying the gel pieces in a Speedvac, samples were reduced with 10 mM dithiothreitol (DTT) in 25 mM NH_4_HCO_3_ at 56°C for 1 h. The supernatant was removed again, and samples were alkylated with 55 mM iodoacetamide (IAA) for 45 min in the dark at room temperature. Gel slices were vortexed with 100 μL 25 mM NH_4_HCO_3_ and then 25 mM NH_4_HCO_3_/50% acetonitrile to wash any remaining DTT and IAA and dehydrate them before being completely dried by Speedvac. The dried gel pieces were overlaid with 0.10 μg/μL sequencing-grade trypsin (Promega) in 25 mM NH_4_HCO_3_, rehydrated on ice for 10 min, and then incubated at 37°C overnight to digest the samples. Digested peptides were extracted from the gel slices three times by vortexing them with 30 μL 50% acetonitrile/5% formic acid solution for 30 min. Extracted digests were combined and reduced to ~10 μL by Speedvac; samples for MALDI-MS analysis were cleaned up using C18 ZipTips (EMD Millipore) and eluted with 50% acetonitrile/0.1% trifluoroacetic acid (TFA).

**MALDI- and LC-MS analysis of tryptic peptides.** The desalted trypsin digest was co-crystallized with matrix on a ground steel target plate with a saturated solution of α-Cyano-4-hydroxycinnamic acid (HCCA) matrix prepared in 50 % acetonitrile with 0.1% trifluoroacetic acid. The samples were then analyzed using both linear modes and reflectron mode on a Bruker Ultraflextreme MALDI-TOF/TOF mass spectrometer.

The LC-MS/MS analysis was performed on a Bruker Impact II q-TOF mass spectrometer coupled to a 1290 uHPLC. The HPLC column used was a Waters XBridge Peptide BEH C18  1mm internal diameter, 300Angsrom pore size, 5 um particle size, 100 mm length was used. Mobile phases were prepared using HPLC grade solvents: A: 0.1% formic acid in water, B: 0.1% formic acid in acetonitrile. A constant flow rate of 0.100 ml/minute was maintained throughout the analysis. An injection volume of 10 uL was used for each sample. The mobile phase composition was started at 3%B and maintained for the first 2 minutes after injection. The %B was increased linearly to 55% over the next 28 minutes to elute most of the peptides. The %B was increased to 70% over the next 5 minutes. Finally, the column was re-equilibrated to starting conditions over 9.9 minutes. Each analysis was internally calibrated using sodium formate clusters to get better than 5 ppm mass accuracy for the run for both the MS and MS/MS data by using a sodium formate solution. The accurate m/z of expected peptide ions were plotted as extracted ion currents in Bruker DataAnalysis software with a m/z window of 0.005 Da and manually annotated. The data-dependent MS/MS data was exported to Mascot Generic Format (.mgf) and searched with Mascot (vs. 2.4, Matrix Science Limited) to confirm identification of non-crosslinked peptides when possible.

**Expression and purification of LptBFGC complexes for ATPase assay.** NR2761 [Δ*lptFG*::*frt* (pBAD18LptFG3)] and NR3327 [Δ*lptFG*::*frt* (pBAD18LptFG3/ LptFE84A/LptGE88A)] were each transformed with (separately) pCDFduet-LptB-LptC-His7 and pCDFduet-LptB(R91S)-LptC-His7. Overnight cultures of NR2761 derivatives were grown in LB supplemented with 50 μg/mL carbenicillin and 50 μg/mL spectinomycin; NR3327 derivatives were grown in LB additionally supplemented with 20 μM IPTG. Overnight cultures were diluted into 3 L fresh media with the same supplements, grown at 30°C to OD_600_ ~0.8, induced by addition of 0.01% (wt/vol) L-arabinose and 500 μM IPTG; temperature was either cooled to 24°C for 10 h expression or increased to 37°C for 3 h expression. Prior to harvesting cells after expression, a 5-mL culture was removed to isolate plasmids so that the *lptB*, *lptF*, and *lptG* genes could later be sequenced to ensure suppressor mutations had not occurred. Cells were lysed, membranes harvested, and LptBFG-LptC-His complexes isolated following the protocol described above for LptB^F90^*^p^*^BPA^FG-LptC-His. ATPase activity was measured with 0.2 μM LptBFGC, 5 mM ATP, and 5 mM MgCl_2_ as described previously (12).

**REFERENCES**

1. Ruiz N, Gronenberg LS, Kahne D, & Silhavy TJ (2008) Identification of two inner-membrane proteins required for the transport of lipopolysaccharide to the outer membrane of *Escherichia coli*. *Proceedings of the National Academy of Sciences of the United States of America* 105:5537-5542.

2. Sherman DJ*, et al.* (2014) Decoupling catalytic activity from biological function of the ATPase that powers lipopolysaccharide transport. *Proceedings of the National Academy of Sciences of the United States of America* 111:4982-4987.

3. Silhavy TJ, Berman ML, & Enquist LW (1984) *Experiments with Gene Fusions* (Cold Spring Harbor Lab Press, Cold Spring Harbor, NY).

4. Wu T*, et al.* (2006) Identification of a protein complex that assembles lipopolysaccharide in the outer membrane of *Escherichia coli*. *Proceedings of the National Academy of Sciences of the United States of America* 103:11754-11759.

5. Freinkman E, Chng S-s, & Kahne D (2011) The complex that inserts lipopolysaccharide into the bacterial outer membrane forms a two-protein plug-and-barrel. *Proceedings of the National Academy of Sciences of the United States of America* 108:2486-2491.

6. de Boer Pa, Crossley RE, & Rothfield LI (1989) A division inhibitor and a topological specificity factor coded for by the minicell locus determine proper placement of the division septum in *E. coli*. *Cell* 56:641-649.

7. Datsenko KA & Wanner BL (2000) One-step inactivation of chromosomal genes in *Escherichia coli* K-12 using PCR products. *Proc Natl Acad Sci U S A* 97(12):6640-6645.

8. Yu D*, et al.* (2000) An efficient recombination system for chromosome engineering in *Escherichia coli*. *Proc Natl Acad Sci U S A* 97(11):5978-5983.

9. Selzer G, Som T, Itoh T, & Tomizawa J (1983) The origin of replication of plasmid p15A and comparative studies on the nucleotide sequences around the origin of related plasmids. *Cell* 32(1):119-129.

10. Ruiz N, Wu T, Kahne D, & Silhavy TJ (2006) Probing the Barrier Function of the Outer Membrane with Chemical Conditionality. *ACS Chemical Biology* 1:385-395.

11. Sampson BA, Misra R, & Benson SA (1989) Identification and characterization of a new gene of *Escherichia coli* K-12 involved in outer-membrane permeability. *Genetics* 122:491-501.

12. Sherman DJ, Okuda S, Denny WA, & Kahne D (2013) Validation of inhibitors of an ABC transporter required to transport lipopolysaccharide to the cell surface in Escherichia coli. *Bioorg Med Chem* 21(16):4846-4851.

13. Casadaban MJ (1976) Transposition and fusion of the lac genes to selected promoters in Escherichia coli using bacteriophage lambda and Mu. *Journal of Molecular Biology* 104(3):541-555.

14. Larkin MA*, et al.* (2007) Clustal W and Clustal X version 2.0. *Bioinformatics* 23(21):2947-2948.

15. Thompson JD, Higgins DG, & Gibson TJ (1994) CLUSTAL W: improving the sensitivity of progressive multiple sequence alignment through sequence weighting, position-specific gap penalties and weight matrix choice. *Nucleic Acids Res* 22(22):4673-4680.

16. Waterhouse AM, Procter JB, Martin DM, Clamp M, & Barton GJ (2009) Jalview Version 2--a multiple sequence alignment editor and analysis workbench. *Bioinformatics* 25(9):1189-1191.

17. Yachdav G*, et al.* (2014) PredictProtein--an open resource for online prediction of protein structural and functional features. *Nucleic Acids Res* 42(Web Server issue):W337-343.

**FIGURE LEGENDS**

**FIG S1. Structure-function analysis of LptB groove variants.** (*A*) OM- permeability defects of haploid strains carrying mutant *lptB* alleles in pET23/42LptB were assessed by disc diffusion assay with four antibiotics. Representative data set for LptB variants that have increased sensitivity (≥3 mm) to at least one antibiotic. Variants not listed reproducibly had no increased sensitivity (≤3 mm) with respect to strain NR2101, which expresses the wild-type *lptB^+^* allele from pET23/42LptB. Data shown is representative of at least three independent experiments. The diameter (in mm) of zones with no growth is noted by numbers and with reduced growth is noted by numbers in parentheses; no visible zone of inhibition is noted as less than the diameter of the disc (6 mm). (*B*) LptB immunoblot showing protein levels of defective LptB variants in cultures grown overnight in rich (LB) or minimal (M63gluc) media. 754 refers to NR754, the wild-type strain expressing *lptB* from its native chromosomal locus (1, 13). For haploid *lptB* strains, WT refers to strain NR2101, which carries pET23/42LptB^WT^. For merodiploid *lptB* strains, WT refers to strain NR2583, which produced LptB^WT^ from both chromosomal *lptB* and pET23/42LptB. As described in Materials and Methods, samples were normalized by the OD_600_ of their cultures.

**FIG S2. Conservation of LptB residues** ClustalW (14, 15) alignment of LptB homologs with groove-exposed residues investigated in this study underlined with red bars. Residues F90, L93, and R150 in *E. coli* LptB are marked with yellow asterisks. Colored in Jalview (16) with percentage identity color scheme: purple = 50 (light) – 100 (dark) %ID, white<50 %ID. Homologs were identified from *Caulobacter crescentus* NA1000 (α-Proteobacteria), *Neisseria meningitidis* Fam18 (β-Proteobacteria), *Escherichia coli* K12 substr. MG1655 (γ-Proteobacteria), *Geobacter uraniireducens* Rfr (δ-Proteobacteria), *Campylobacter jejuni* RM1221 (ε-Proteobacteria), *Gramella forsetii* KT0803 (Bacteroidetes), *Elusimicrobium minutum* Pei191(Elusimicrobia), *Sulfurihydrogenibium azorense* Az-Fu1 (Aquificae), *Denitrovibrio acetiphilus* DSM 12809 (Deferribacteres), and *Anabaena variabilis* ATCC29413 (Cyanobacteria).

**FIG S3. *p*Bpa-containing LptB and LptFG variants that do not show direct interaction with partners.** (*A*) Heightened exposure and contrast of LptB immunoblot from Fig 1C showing low efficiency crosslinks from R92*p*Bpa and Q104*p*Bpa (top panel: exposure = 250 s and contrast high = 20,000; middle panel: exposure = 150 s and contrast high = 52,000). (*B*) LptB immunoblot showing LptB variants containing *p*BPA substitutions that do not yield detectable UV-dependent cross-links. WT refers to strain NR3877, which contains no *p*BPA substitutions in pET23/42LptB. (*C-D*) LptB immunoblot of LptF-*p*BPA (*C*) and LptG-*p*BPA (*D*) variants that do not yield detectable UV-dependent cross-links to LptB. WT refers to strain NR3720, which contains no *p*BPA substitutions in pBAD18LptFG3.

**FIG S4. LptB^F90^*^p^*^Bpa^ cross-links to LptFGB.** LptB (left), LptF (center) and LptG (right) immunoblots of strain NR3540 carrying pCL-His_6_-LptB^F90^*^p^*^BPA^ and pBAD18LptFG3 to increase levels of LptFG. WT refers to strain NR3720, which contains no *p*BPA substitutions in pCL-His_6_-LptBl. The LptB^F90^*^p^*^Bpa^- LptF and LptG cross-links are recognized by antisera raised against LptB, LptF and LptG, and marked B-XL, F-XL and G-XL, respectively.

**FIG S5. Alignment of distant LptF and LptG homologs.** ClustalW (14, 15) alignment shows six conserved, highly-hydrophobic regions that correlate with transmembrane domains of *E. coli* LptF (marked with orange lines) and LptG (marked with purple lines) predicted by TMSEG (Predictprotein) (17). Homologs were identified from *Caulobacter crescentus* NA1000 (α-Proteobacteria), *Neisseria meningitidis* Fam18 (β-Proteobacteria), *Escherichia coli* K12 substr. MG1655 (γ-Proteobacteria), *Geobacter uraniireducens* Rfr (δ-Proteobacteria), *Campylobacter jejuni* RM1221 (ε-Proteobacteria), *Gramella forsetii* KT0803 (Bacteroidetes), *Elusimicrobium minutum* Pei191(Elusimicrobia), *Sulfurihydrogenibium azorense* Az-Fu1 (Aquificae), *Denitrovibrio acetiphilus* DSM 12809 (Deferribacteres), and *Anabaena variabilis* ATCC29413 (Cyanobacteria). Colored in Jalview (16) with clustalx color scheme: light blue = hydrophobic, green = hydrophilic, orange = Gly, yellow = Pro, magenta = negatively charge, red = positively charged.

**FIG S6. Identification of cross-link containing peptides in LptB^F90^*^p^*^BPA^-LptF adducts via MALDI and LC-MS/MS.** (*A*) Table showing tryptic digest peptides of LptB^F90^*^p^*^BPA^ and LptF which are part of the cross-linked peptide described in figure 2, listed with the monoisotopic mass of each neutral peptide or modification. Residues where trypsin failed to cleave the cross-link adduct appear in red. The predicted mass of the cross-linked adduct is the sum of the species highlighted in blue. (*B*) MALDI-TOF traces for trypsin-digest LptB^F90^*^p^*^BPA^ (bottom panel) and LptB^F90^*^p^*^BPA^-LptF adduct (top panel) in m/z range 1550-1560. From trypsin-digested LptB^F90^*^p^*^BPA^, we expected to see peptide 79-GIGYLPQEASI(F90*p*BPA)R-91 at [M+H] = 1554.8919. As expected this was observed in the uncross-linked sample but not in the cross-linked sample, presumably because *p*BPA has crosslinked something. The overlapping peak at m/z = 1552.882 could not be identified and is likely a contaminant; notably its abundance is roughly equal between the two samples whereas the abundance of the m/z =1554.8919 peak decreases greatly. (*C*) The LptF peptide 96 – AVLVK – 100 is adjacent to the coupling helix and has theoretical [M+H] 529.3708. The two panels show intensity vs. time for the m/z range 529.3708±0.005 for the LptF (top) and LptB^F90^*^p^*^BPA^-LptF adduct (bottom) samples run on LC-MS. (*D*) Shows detected masses in the range of 6.7-7.2 minutes in the LptF sample. The high peak at 529.3717 is within the error of the detector for the correct mass for the LptF peptide AVLVK. (*E*) To detect larger, potentially cross-linked peptides, low resolution analysis of digested LptB^F90^*^p^*^BPA^, LptF, LptG, and LptB^F90^*^p^*^BPA^-LptF adduct was performed using MALDI-TOF in linear mode with the m/z range expanded. A unique peak is present in the LptB^F90^*^p^*^BPA^-LptF sample, which we hypothesize is an adduct of LptB^F90^*^p^*^BPA^(79-92) with LptF(96- 126).

**FIG S7. Structure-function analysis of LptFG coupling helix variants.** (*A*) OM permeability of haploid *lptFG* mutants measured by disc diffusion assay of four antibiotics as described in Fig. S1A. Strains carry *lptFG* alleles on pBAD18LptFG3 derivatives. NR2761 was used as the wild-type *lptFG^+^* control. Sequence of the LptFG coupling helices is shown above. (*B*) Protein levels of defective LptFG variants in cultures grown overnight. LptF and LptG immunoblots of haploid strains for partial loss-of-function LptF (left) and LptG (right) variants, respectively, grown in LB. (*C*) LptF and LptG immunoblot of merodiploid strains for total loss-of-function variants grown in LB and of haploid strains for conditional loss-of-function variants grown in glucose minimal medium. 754 refers to strain NR754, which was used as the control for chromosomally produced LptFG. For haploid strains, WT refers to NR2761. For merodiplid strains, WT refers to NR3079.
