# Supplementary material for: Identification of Residues in the Lipopolysaccharide ABC Transporter That Coordinate ATPase Activity with Extractor Function
Source: mBio. 2016 Oct 18;7(5):e01729-16. doi: 10.1128/mBio.01729-16 (PMC5082905; doi:10.1128/mBio.01729-16)
Supplement: Figure S5 — Alignment of distant LptF and LptG homologs. ClustalW (M. A. Larkin, G. Blackshields, N. P. Brown, R. Chenna, P. A. McGettigan, H. McWilliam, F. Valentin, I. M. Wallace, A. Wilm, R. Lopez, J. D. Thompson, T. J. Gibson, and D. G. Higgins, Bioinformatics 23:2947–2948, 2007; J. D. Thompson, D. G. Higgins, and T. J. Gibson, Nucleic Acids Res 22:4673–4680, 1994) alignment shows six conserved, highly hydrophobic regions that correlate with transmembrane domains of E. coli LptF (marked with orange lines) and LptG (marked with purple lines) predicted by TMSEG (Predictprotein) (G. Yachdav G, Kloppmann E, L. Kajan, M. Hecht, T. Goldberg, T. Hamp, P. Honigschmid, A. Schafferhans, M. Roos, M. Bernhofer, L. Richter, H. Ashkenazy, M. Punta, A. Schlessinger, Y. Bromberg, Schneider R, G. Vriend, C. Sander, N. Ben-Tal, and B. Rost, Nucleic Acids Res 42:W337–W343, 2014). Homologs from Caulobacter crescentus NA1000 (Alphaproteobacteria), Neisseria meningitidis Fam18 (Betaproteobacteria), Escherichia coli K-12 strain MG1655 (Gammaproteobacteria), Geobacter uraniireducens Rfr (Deltaproteobacteria), Campylobacter jejuni RM1221 (Epsilonproteobacteria), Gramella forsetii KT0803 (Bacteroidetes), Elusimicrobium minutum Pei191 (Elusimicrobia), Sulfurihydrogenibium azorense Az-Fu1 (Aquificae), Denitrovibrio acetiphilus DSM 12809 (Deferribacteres), and Anabaena variabilis ATCC 29413 (Cyanobacteria) were identified. Coloring was done in Jalview (A. M. Waterhouse, J. B. Procter, D. M. Martin, M. Clamp, and G. J. Barton, Bioinformatics 25:1189–1191, 2009) with the following ClustalX color scheme: light blue, hydrophobic; green, hydrophilic; orange, Gly; yellow, Pro; magenta, negatively charged; red, positively charged. Download [file mbo005163035sf5.pdf]

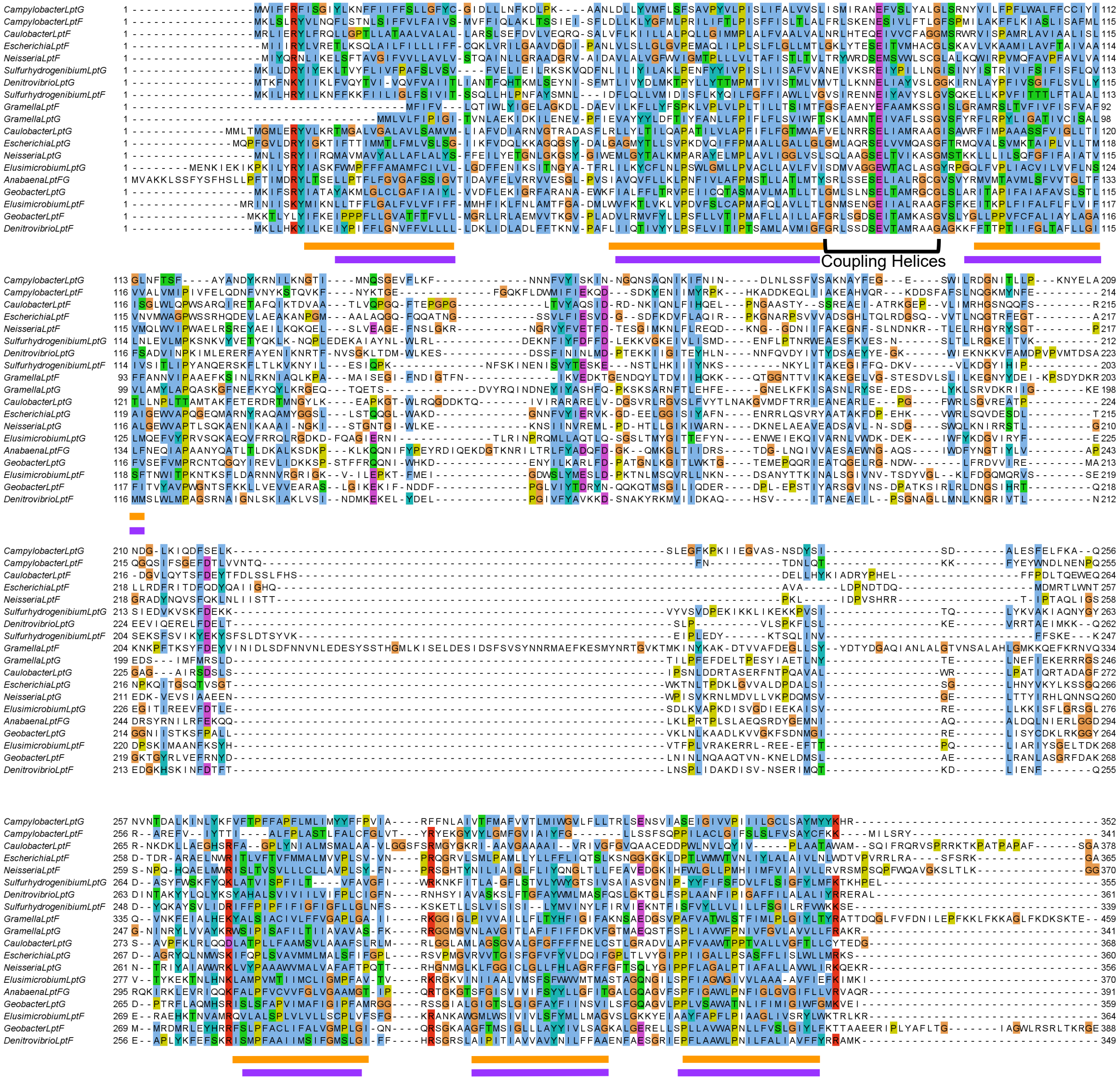
CampylobacterLptG
CampylobacterLptF
CaulobacterLptF
EscherichiaLptF
NeisseriaLptF
SulfurhydrogenibiumLptG
DenitrovibrioLptG
SulfurhydrogenibiumLptF
GramellaLptF
GramellaLptG
CaulobacterLptG
EscherichiaLptG
NeisseriaLptG
ElusimicrobiumLptG
AnabaenaLptFG
GeobacterLptG
ElusimicrobiumLptF
GeobacterLptF
DenitrovibrioLptF
Coupling Helices

FIG S5
